# Support‐Free, Connected Core–Shell Nanoparticle Catalysts Synthesized via a Low‐Temperature Process for Advanced Oxygen Reduction Performance

**DOI:** 10.1002/advs.202408614

**Published:** 2024-12-26

**Authors:** Aparna Chitra Sudheer, Gopinathan M. Anilkumar, Hidenori Kuroki, Takeo Yamaguchi

**Affiliations:** ^1^ Laboratory for Chemistry and Life Sciences Tokyo Institute of Technology Yokohama Kanagawa 226–8501 Japan; ^2^ R&D Center Noritake Co., Ltd 300 Higashiyama Miyoshi‐Cho Aichi 470‐0293 Japan

**Keywords:** connected core–shell nanoparticles, oxygen reduction reaction, polymer electrolyte fuel cell, Pt shell, support‐free catalyst

## Abstract

Nanostructured Pt‐based catalysts have attracted considerable attention for fuel‐cell applications. This study introduces a novel one‐pot and low‐temperature polyol approach for synthesizing support‐free, connected nanoparticles with non‐Pt metal cores and Pt shells. Unlike conventional heat treatment methods, the developed support‐free and Fe‐free connected Pd_core_@Pt_shell_ (Pd@Pt) nanoparticle catalyst possesses a stable nanonetwork structure with a high surface area. This approach can precisely control the atomic‐level structure of the Pt shell on the Pd core at a low deposition temperature. The optimized Pd@Pt catalyst with a Pt/Pd atomic ratio of 0.8 and a Pt shell thickness of 1.1 nm exhibits a threefold improvement in oxygen reduction reaction (ORR) mass activity compared to that of commercial carbon‐supported Pt nanoparticle catalyst (Pt/C). Durability evaluation demonstrated 100% retention of specific activity after 10,000 load cycles, owing to the stable nanonetwork and uniform coverage of the Pt shell. In addition, the support‐free, connected core–shell nanoparticle catalyst overcomes the carbon corrosion issues commonly associated with conventional carbon‐supported catalysts while simultaneously improving both ORR activity and load cycle durability. These findings highlight the potential of this innovative approach to develop support‐free catalysts for polymer electrolyte fuel cells and other energy devices.

## Graphical abstract

A novel approach for synthesizing support‐free, connected nanoparticles with non‐Pt metal core and Pt atomic shell is presented. It employs a low‐temperature synthesis method, enabling significant improvements in the catalyst surface area and oxygen reduction reaction performance. The support‐free, connected Pd_core_@Pt_shell_ catalyst demonstrated high structural stability with 100% specific activity retention even after 10,000 potential cycles at an elevated temperature.

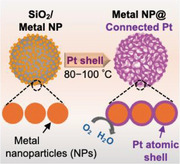

## 1 Introduction

In recent years, polymer electrolyte fuel cells (PEFCs) have emerged as a highly promising renewable energy solution to address the current global energy crisis and pressing environmental challenges.^[^ ^1^ ^]^ Pt nanoparticles supported on conductive carbon (Pt/C) have been conventionally used to enhance the kinetics of the sluggish oxygen reduction reaction (ORR) at the cathode.^[^ ^2^ ^]^ However, a substantial amount of platinum group metals (PGM) is required, which constitutes ≈41% of the total costs of fuel cell stacks.^[^ ^3^ ^]^ Moreover, during start‐stop operation, the carbon supports tend to degrade, causing the aggregation and detachment of Pt particles, which gradually reduces catalytic activity over time.^[^ ^4^ ^]^ In summary, the commercial application of PEFCs is hindered by the high material cost, carbon corrosion, insufficient ORR activity, and the compromised durability of conventional Pt‐based nanoparticle catalysts on carbon supports.^[^ ^4^, ^5^, ^6^ ^]^ To address these challenges, numerous researchers have attempted to modify the structure of electrocatalysts to reduce Pt usage and enhance catalytic activity. The surface structure, size, shape, and composition of Pt nanoparticles can affect the Pt−Pt bond distance, Pt 5*d* vacancy, Pt coordination number, and *d*‐band center, thereby influencing their intrinsic catalytic properties.^[^ ^7^, ^8^, ^9^, ^10^ ^]^ Effective ORR catalysts have been developed with different materials (Pt alloys,^[^ ^11^, ^12^ ^]^ multi‐metallic high‐entropy alloys,^[^ ^13^, ^14^, ^15^, ^16^ ^]^ core–shell,^[^ ^17^, ^18^ ^]^ metal chalcogenides,^[^ ^19^ ^]^ precious metal‐free materials,^[^ ^20^, ^21^, ^22^ ^]^ single‐atom catalysts (SACs),^[^ ^23^ ^]^ single‐site catalysts (SSCs),^[^ ^24^ ^]^ etc.) and various shape‐controlled structures (nanoframes,^[^ ^25^, ^26^ ^]^ nanocylinders,^[^ ^27^ ^]^ nanowires,^[^ ^28^, ^29^, ^30^, ^31^ ^]^ nanotubes,^[^ ^32^, ^33^ ^]^ nanosheets/plates,^[^ ^34^, ^35^ ^]^ nanocages,^[^ ^36^ ^]^ nano/meso structured thin films,^[^ ^37^ ^]^ etc.). Especially, carbon‐free nanostructured Pt‐based catalysts have gained widespread attention for tackling carbon corrosion issues while improving catalytic activity and durability.^[^ ^38^, ^39^, ^40^, ^41^, ^42^ ^]^

**Scheme 1:**
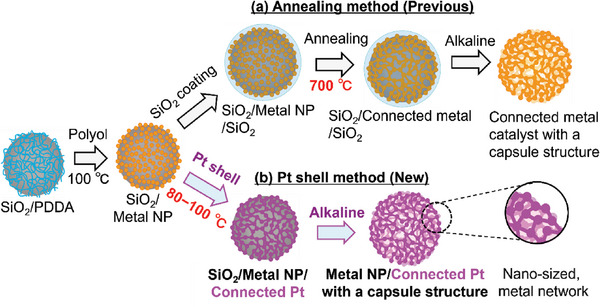
Synthesis of connected nanonetwork catalysts with a porous hollow capsule structure. a) Conventional method using SiO_2_ coating and high‐temperature annealing treatment, resulting in a connected metal (M) catalyst with a large crystallite size. b) Pt shell method using a low‐temperature process to obtain a connected core–shell catalyst with a small crystallite size.

Previously, our group reported connected platinum−iron (Pt−Fe) nanoparticle catalysts featuring a porous, hollow capsule structure, which eliminated the need for conducting support materials like carbon.^[^ ^43^ ^]^ This unique structure forms a highly conductive network, and the ORR specific activity (ORR activity per unit Pt surface area) was enhanced by ninefold compared to that of commercial Pt/C. We also demonstrated that the connected Pt–Fe alloy nanoparticle catalyst showed threefold higher ORR specific activity than that of Pt–Fe alloy nanoparticles (without particle's connection) on carbon black. This carbon‐free connected structure exhibits a moderate interaction with O species and suppresses Pt oxidation poisoning by water molecule, which contributed to the enhanced ORR durability.^[^ ^44^ ^]^ On the other hand, optimizing the membrane–electrode assembly (MEA) performance of a new material involves numerous factors beyond the catalyst itself, such as interface structure, ionomer properties and morphologies, thickness and pore structures of catalyst layers, and operation conditions. In this regard, we have demonstrated the effect of ionomer morphology in carbon‐free catalyst layers with connected Pt–Fe alloy nanoparticle catalysts, suggesting the importance of a uniform, thin ionomer coating to achieve practical fuel cell performance.^[^ ^45^ ^]^ Furthermore, the MEA fabricated using connected Pt−Fe catalysts showed a stable electrochemically active surface area (ECSA) after 10,000 start‐stop cycles, while Pt/C experienced a drastic ECSA decrease due to carbon corrosion.^[^ ^43^ ^]^ The enhanced ORR activity and durability of the connected nanoparticle materials and their practical fuel cell performance underscore their significance in diverse fuel cell applications. As shown in **Scheme** **1**a, to develop connected networks between nanoparticles, high‐temperature and high‐pressure supercritical treatment (300 °C and 20 MPa)^[^ ^43^ ^]^ or high‐temperature annealing (500–700 °C and ambient pressure)^[^ ^46^ ^]^ are essential. However, annealing at 700 °C significantly increases the particle size (≈17.9 nm) of the connected Pt−Fe catalyst, which reduces the number of active sites and leads to a sevenfold decrease in the ECSA compared to that of commercial Pt/C with a particle size of 2–3 nm. Moreover, the presence of Fe ions can promote the formation of OH radicals, causing the degradation of the ionomer and polymer electrolyte membranes.^[^ ^47^ ^]^ Hence, utilizing a Fe‐free catalyst and applying a low‐temperature treatment are favorable strategies to enhance the activity and durability of support‐free catalysts.

In this study, we report a novel one‐pot synthesis method using a two‐step polyol process for developing connected Pt‐based core–shell nanoparticle catalysts without any high‐temperature treatment, as illustrated in Scheme 1b. The resulting nanoparticles formed a stable nanonetwork through the connection of Pt shell. The carbon‐support‐free porous hollow capsule structure enabled the formation of a thin and highly porous catalyst layer (porosity >80%), which was beneficial for oxygen mass transport.^[^ ^43^ ^]^ Thus, due to the above structural advantages, the developed catalyst has the potential to exhibit high ORR activity, enhanced durability, and improved performance as a PEFC catalyst.

A variety of metals, such as Au, Ir, Ru, Rh, Pd, Ni, Cu, and Co, have been used as cores with Pt shells on carbon supports, and these core–shell nanoparticle catalysts aim to enhance ORR performance while minimizing Pt usage.^[^ ^17^, ^18^, ^48^, ^49^, ^50^, ^51^ ^]^ In particular, Pd has garnered significant interest as a core metal because Pt monolayers exhibit superior ORR‐specific activity on Pd (111). This enhancement is attributed to electron transfer from Pd to the modified Pt surface and the lattice contraction of Pt, which shifts the *d*‐band center of the Pt surface, as evidenced by relevant computational investigations.^[^ ^52^, ^53^, ^54^, ^55^, ^56^, ^57^, ^58^ ^]^ Previous studies on Pd_core_@Pt_shell_ (Pd@Pt) core–shell nanoparticles also revealed a notable dependence of the ORR performance on the Pt shell thickness.^[^ ^55^, ^59^, ^60^ ^]^

In this study, we utilized Pd as the core nanoparticle to explore the impact of a connected Pt shell on the ORR activity. Employing our novel synthesis route, a support‐free and Fe‐free core–shell nanonetwork catalyst was synthesized featuring Pd@Pt. The atomic‐level structure of the Pt shell was precisely controlled with the aim of investigating the relationship between the Pt shell thickness and ORR activity. The successful synthesis of a support‐free connected Pd@Pt catalyst synthesized using the one‐pot polyol method possessed a stable network structure with a high surface area. Comprehensive characterization using various techniques provided insights into the properties of the connected Pd@Pt catalysts. Furthermore, the developed catalyst exhibits exceptional durability, robust performance, and structural resilience under repeated load cycles.

## 2 Results and Discussion

### 2.1 Synthesis and Structural Analysis of the Connected Pd@Pt Core–Shell Catalyst

Previously reported, connected nanoparticle catalysts were prepared by depositing metal nanoparticles onto a spherical SiO_2_ template followed by coating with a SiO_2_ layer and annealing at high temperatures (500–700 °C), as depicted in Scheme 1a.^[^ ^46^ ^]^ However, this method involves thermal fusion and leads to significant particle growth and a decrease in ECSA. To address these issues, we developed a novel one‐pot polyol process at a low temperature (≈100 °C) to synthesize connected Pd core‐Pt shell catalysts with high‐surface‐areas, as illustrated in Scheme 1b. In this method, Pd nanoparticles were first deposited on a silica template by polyol process (Pd/SiO_2_). Then, Pt shell was formed on Pd/SiO_2_ using a controlled polyol method, resulting in a connected Pd@Pt nanonetwork. For depositing monolayers or sub‐monolayers of Pt onto Pd nanocrystals, previous studies have explored electrochemical approaches by leveraging the galvanic replacement reaction between an underpotentially deposited Cu monolayer and a Pt(II) precursor.^[^ ^61^ ^]^ However, the scalability of this process is greatly limited by the involvement of electrodes and the low yield of the catalyst (only several tens of micrograms). In our study, we employed a versatile one‐pot polyol method to introduce a Pt shell, wherein the Pt precursor was carefully injected into the Pd core solution at a low temperature (≈100 °C). This approach not only allowed large‐scale synthesis but also enabled precise control of the shell formation. The shell structures were controlled by adjusting the temperature (80–100 °C), reaction time (3–20 h), and injection rate (0.02–1 mL min^−1^). The detailed procedure for synthesizing the connected Pd@Pt core–shell catalysts with varying Pt/Pd ratios and their structural analysis methods are provided in the experimental section of the Supplementary Information.

The compositions of the prepared catalysts were estimated from the inductively coupled plasma atomic emission spectroscopy (ICP−AES) results. Using the different conditions for Pt shell formation, the catalysts with different Pt coverages, as indicated by varying Pt/Pd ratios, were successfully synthesized. The catalyst with Pt/Pd atomic ratios of 0.3, 0.8, and 1.5 are denoted as Pd@Pt_0.3_, Pd@Pt_0.8_, and Pd@Pt_1.5_, respectively.

**Figure 1:**
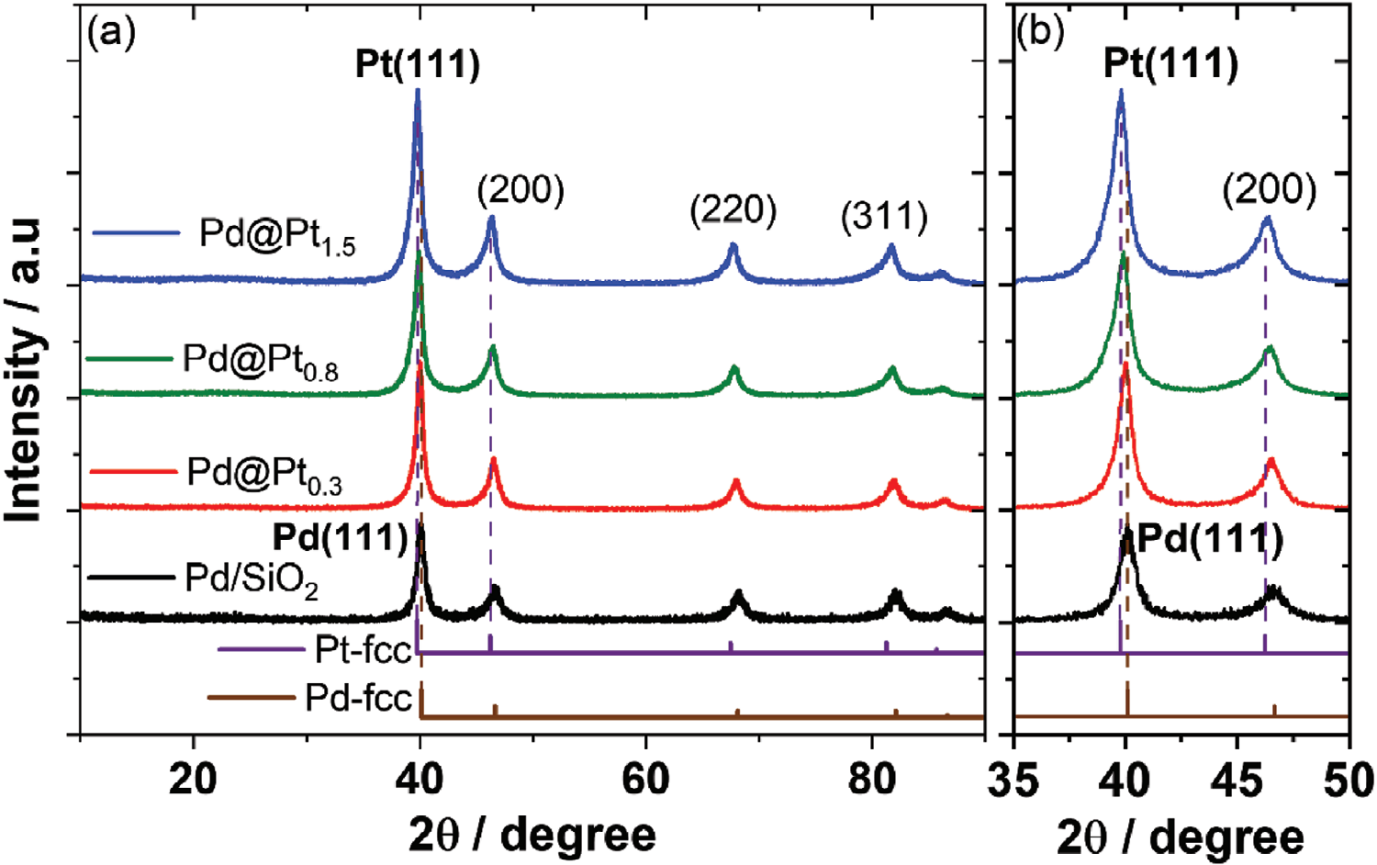
XRD patterns of the Pd/SiO_2_ and Pd@Pt catalysts.

The X‐ray diffraction (XRD) patterns (**Figure** **1**a) demonstrate that the Pd/SiO_2_ catalyst shows a peak at the two theta value of 40.10°, which corresponds to the (111) plane of Pd in the fcc phase (ICSD database 00‐005‐0681). After the formation of Pt shells, Pd@Pt_0.3_, Pd@Pt_0.8_, and Pd@Pt_1.5_ catalysts show peaks at lower two theta values of 39.98°, 39.90°, and 39.62°, respectively, corresponding to the (111) plane of the Pt in the fcc phase (ICSD database 00‐004‐0802). The crystallite size of the Pd/SiO_2_ catalyst is 8.0 ± 1.0 nm. After Pt deposition, the crystallite sizes of the Pd@Pt_0.3_, Pd@Pt_0.8_, and Pd@Pt_1.5_ catalysts increase to 8.5 ± 1.0, 10.0 ± 1.0, and 14.0 ± 2.0 nm, respectively (Table S1, Supporting Information). Based on the ICP‒AES data, the thickness of a Pt monolayer was estimated by assuming a conformally uniform Pt shell on the Pd nanoparticles. Given that the average diameter of the Pd nanoparticles is 8.0 ± 1.0 nm, as determined via XRD analysis,^[^ ^62^ ^]^ the shell thicknesses of Pd@Pt_0.3_, Pd@Pt_0.8_, and Pd@Pt_1.5_ are ≈1.0, 3.5, and 6.0 monolayers, respectively.

**Figure 2:**
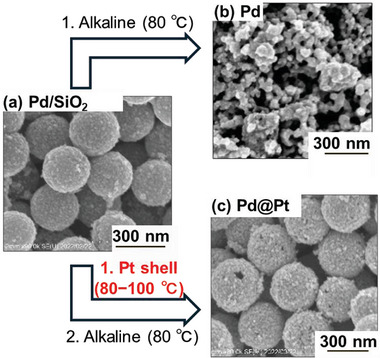
SEM images of a) Pd nanoparticles uniformly deposited on a silica template (Pd/SiO_2_ catalyst), b) Pd catalyst obtained by removing the silica template from the Pd/SiO_2_ catalyst via alkaline treatment, and c) Pd@Pt catalyst obtained by forming Pt shells on Pd/SiO_2_ catalyst followed by removing silica template via alkaline treatment.

The scanning electron microscopy (SEM) image (**Figure** **2**a) suggests the uniform distribution of Pd nanoparticles on the silica template (Pd/SiO_2_) after the polyol reaction. Figure 2b shows an image of the Pd catalyst after removing SiO_2_ via alkaline treatment. The spherical structure of the catalyst collapses after removing the template, and the Pd nanoparticles agglomerate, indicating an unstable nanoparticle network. To address this issue, a Pt shell was introduced on Pd/SiO_2_ (Scheme 1b). Subsequently, the silica template was removed by alkaline treatment, resulting in a stable connected network consisting of a Pd catalyst with a connected Pt shell (Pd@Pt) (Figure 2c). Notably, the SEM image of the resulting Pd@Pt core–shell catalyst after silica removal demonstrates that the particles maintain their uniform morphology, forming a hollow capsule structure with a connected Pt nanonetwork. This finding emphasizes the crucial role of the Pt shell in ensuring structural stability during template removal.

**Figure 3:**
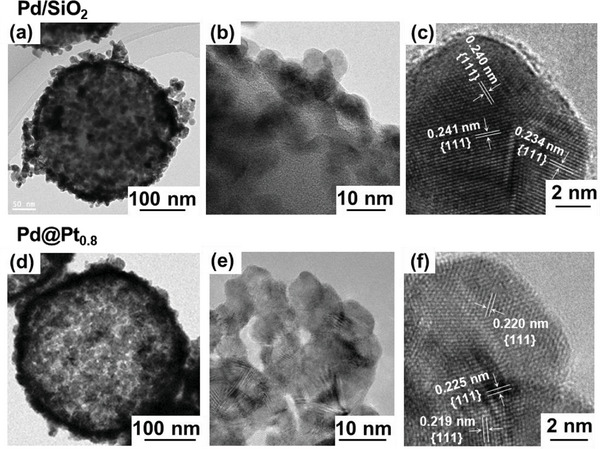
TEM and high resolution‐TEM images of a–c) Pd nanoparticles uniformly deposited on a silica template (Pd/SiO_2_ catalyst) and d–f) Pd/SiO_2_ catalyst after Pt shell formation and silica removal via alkaline treatment (connected Pd@Pt_0.8_ catalyst).

The transmission electron microscopy (TEM) images (**Figure** **3**a–c) show the well‐dispersed Pd nanoparticles on the silica substrate with an average size of 8.0 ± 1.0 nm in accordance with that calculated from the XRD patterns. The high‐magnification TEM image (Figure 3c) reveals the lattice fringes of the Pd nanoparticles, wherein the spacings of 0.240, 0.241, and 0.234 nm correspond to the {111} plane of Pd. The TEM images of the Pd@Pt_0.8_ catalyst (Figure 3d–f) show a uniform distribution of Pt nanoparticles with a connected network structure, confirming the formation of a nanosized network with an average size of 10.0 ± 2.0 nm, which is in accordance with the XRD results. The TEM images of the Pd/SiO_2_ catalyst before and after the formation of the connected Pt network demonstrated that the catalyst maintained its crystallite size throughout the network formation process. This indicates that the synthesis method is effective in creating a connected Pt network while preserving the crystallite size. This observation is particularly important compared to the previously reported Pt–Fe catalysts formed through high‐temperature annealing (700 °C), where the development of connected networks between Pt‐alloy nanoparticles results in a sevenfold increase in the crystallite size.^[^ ^46^ ^]^

As shown in the high‐magnification TEM image (Figure 3f), the lattice spacing of 0.191 nm corresponds to the {200} plane, and those of 0.220, 0.225, and 0.219 nm correspond to the {111} plane of Pt. These values are comparable to the *d*‐spacing of 0.2266 nm for the {111} planes of Pt nanoparticles in commercial Pt/C,^[^ ^55^ ^]^ suggesting that the fcc nanostructure is enclosed by the {111} and {200} facets. The observed decrease in the *d*‐spacing indicates compressive strain in the Pt shell owing to the lattice mismatch between Pt and Pd.^[^ ^55^, ^57^ ^]^ With an average *d*‐spacing of 0.221 nm for the {111} plane, the Pd@Pt_0.8_ catalyst exhibits a 2.5% lattice mismatch relative to Pt/C, resulting in significant compressive strain on the catalyst surface.

**Figure 4:**
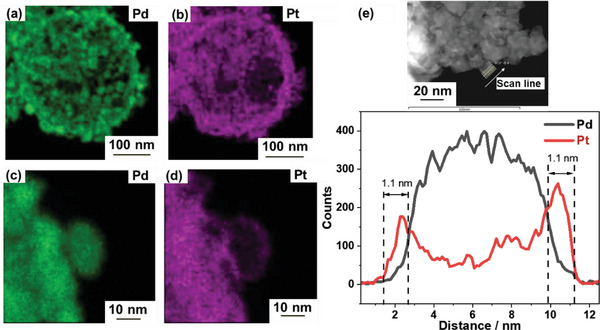
a–d) STEM–EDX elemental mapping images and e) STEM–EDX line scan along the arrow of the connected Pd@Pt_0.8_ catalyst.

The scanning transmission electron microscopy–energy dispersive X‐ray (STEM–EDX) spectroscopy area mapping images (**Figure** **4**a–d) reveal a uniform distribution of Pt and Pd elements, suggesting the presence of a thin Pt shell surrounding the Pd core. These images (Figure 4e) further confirm the core–shell structure with a Pt layer thickness of 1.1 nm. This highlights the successful formation of a stable network structure by connecting the nanoparticles through the Pt shells, thereby maintaining the desired nanosize, as shown in Scheme 1.

**Figure 5:**
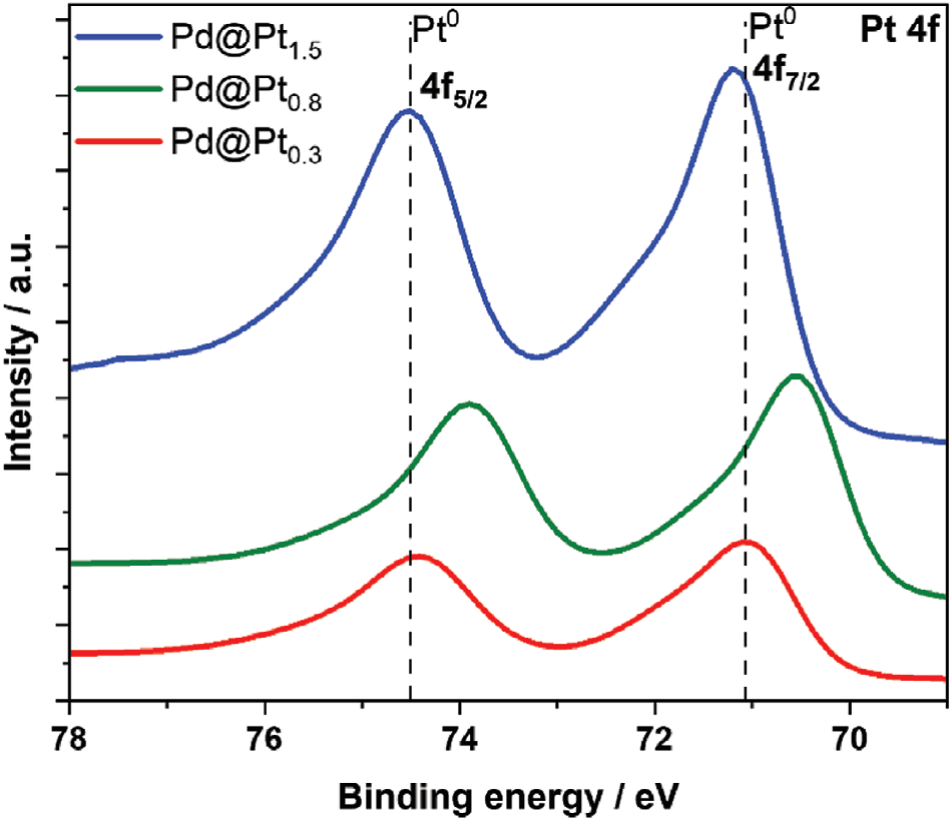
XPS spectra of different connected Pd@Pt catalysts in the Pt 4f region.

XPS analysis (**Figure** **5**) of the Pt 4*f* region shows that the Pt 4*f*_5/2_ peak of standard Pt^0^ (71.2 eV)^[^ ^63^ ^]^ shifts by 0.1 and 0.7 eV for Pd@Pt_0.3_ (71.0 eV) and Pd@Pt_0.8_ (70.5 eV), respectively. A consistent trend is observed for the Pt 4*f*_5/2_ peak, where the positions of Pd@Pt_0.3_ (74.4 eV) and Pd@Pt_0.8_ (73.8 eV) shift by 0.1 and 0.7 eV, respectively, in comparison to the standard elemental Pt^0^ value of 74.5 eV.^[^ ^63^ ^]^ The downward shift in the binding energy suggests the interactions between Pd and Pt, possibly due to the formation of Pt shells around the Pd core and the change in the surface structure due to the formation of a connected network. Pt has a slightly higher electronegativity (2.28) than Pd (2.20); therefore, Pt can withdraw electrons from Pd in the Pd@Pt system.^[^ ^64^ ^]^ The higher electron cloud density around Pt atoms in these catalysts results in an optimal oxygen binding energy for ORR. However, the binding energies of the Pt 4*f*_7/2_ (71.3 eV) and Pt 4*f*_5/2_ (74.6 eV) peaks in Pd@Pt_1.5_ increased by 0.23 and 0.13 eV, respectively. Owing to the increased Pt loading, the Pd@Pt_1.5_ catalyst may exhibit heavy Pt coverage with a continuous, thick, and protective Pt shell. Previous studies have suggested a correlation between higher Pt coverages and increased Pt binding energies, emphasizing the influence of the surface composition on the shifts in binding energy.^[^ ^59^, ^65^ ^]^ Further investigations and in‐depth analyses in future studies are necessary for a comprehensive understanding of the observed shifts in the binding energies. Additional details regarding structural characterization can be found in the experimental section of the Supplementary Information.

### 2.2 ORR Activity of the Connected Pd@Pt Catalysts

To study the effect of the Pt shell on the catalytic performance, cyclic voltammetry (CV) and linear sweep voltammetry (LSV) measurements were recorded for connected Pd@Pt catalysts with Pt/Pd atomic ratios of 0.3, 0.8, and 1.5. Details regarding the electrochemical characterization are provided in the Experimental section of Supplementary Information.

**Figure 6:**
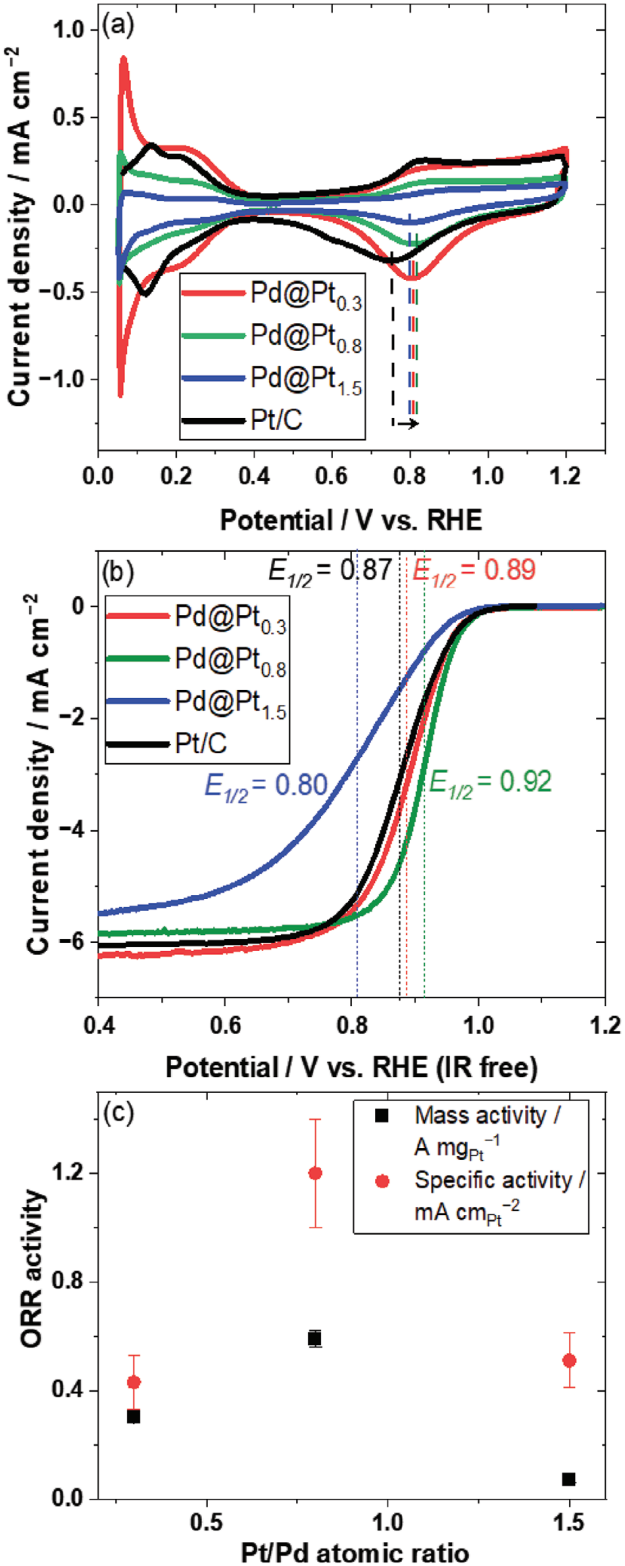
a) CV (N_2_ saturated atmosphere, scan rate: 20 mV s^−1^) and b) LSV curves (O_2_ saturated atmosphere, scan rate: 20 mV s^−1^, scan speed: 1600 rpm) of Pd@Pt catalysts with different Pt/Pd ratios and the commercial Pt/C catalyst. c) Mass activity and specific activity as a function of the Pt/Pd atomic ratio.

**Figure** **6**a,b shows their CV and LSV curves, respectively. In the CV curve of the Pd@Pt_0.3_ catalyst, a sharp peak is observed in the potential range of 0.05–0.1 V, which is attributed to the adsorption and desorption of hydrogen from the Pd core.^68^ This indicates that the Pt shell does not fully cover the Pd core, leaving some Pd atoms exposed on the catalyst surface. The Pd@Pt_0.3_ catalyst shows a high ECSA value of 113 m^2^ g_Pt_^−1^, with a dominant Pd peak in the CV curve, confirming that a significant portion of the Pd core is exposed on the surface. As the Pt/Pd ratio increases, the intensity of the Pd peak decreases significantly. For the Pd@Pt_1.5_ catalyst, the intensity of the Pd peak is almost negligible, indicating a near‐complete coverage of the surface by the Pt‐shell layer.^[^ ^66^ ^]^ At the same time, the ECSA gradually decreases with increasing Pt content, and the ECSA values of the Pd@Pt_0.8_ and Pd@Pt_1.5_ catalysts are 48 ± 3 m^2^ g_Pt_^−1^ and 16 ± 3 m^2^ g_Pt_^−1^, respectively. The Pd@Pt_0.3_ and Pd@Pt_0.8_ catalysts achieve a significantly higher ECSA than the previously reported connected Pt–Fe catalysts (ECSA: 15–30 m^2^ g_Pt_^−1^), which are made through a high‐temperature annealing (500–700 °C) process.^[^ ^46^ ^]^

The CVs of the Pd/C and Pt/C catalysts exhibit similar features, except for the hydrogen absorption/desorption peaks.^[^ ^65^ ^]^The hydrogen desorption peak is crucial for calculating the ECSA, which includes the contribution from the connected Pt surface and the Pd core. This peak appears in the potential range of 0.05–0.1 V and cannot be distinctly separated. Consequently, the ECSA values determined for the connected Pd@Pt catalysts should be considered reference values and may be overestimated.

The LSV curves of the connected Pd@Pt_0.3_ and Pd@Pt_0.8_ catalysts shift to higher potentials compared to that of the Pt/C catalyst, indicating improved ORR activity. Figure 6c compares the electrochemical results of the connected Pd@Pt catalysts with different Pt/Pd atomic ratios. Among the tested catalysts, the Pd@Pt_0.8_ catalyst displays the highest ORR mass activity of 0.59 ± 0.03 A mg_Pt_^−1^ and a specific activity of 1.2 ± 0.2 mA cm_Pt_^−2^. As shown in Figure 6b, its half‐wave potential (*E₁/₂*) reaches 0.92 V, surpassing that of Pt/C and other Pd@Pt catalysts. This improvement can be attributed to a more uniform Pt coverage, which enhances the surface properties of the catalyst.

The low activity of the Pd@Pt_0.3_ catalyst is related to its highly exposed Pd surface, whereas the thick Pt shell in the Pd@Pt_1.5_ catalyst is responsible for its decreased activity. Previous studies have highlighted the potential drawbacks of Pt monolayers, which can degrade during fuel cell operation as transition metals gradually leach out and form thicker Pt overlayers.^[^ ^67^ ^]^ Although a thicker Pt shell may enhance durability, it compromises the beneficial strain and/or ligand effects of the core, in addition to increasing the total PGM loading.^[^ ^67^ ^]^

Therefore, a moderate Pt content on the Pd core, as demonstrated by the connected Pd@Pt_0.8_ catalyst, results in an optimal Pt shell thickness with the highest catalytic performance. Numerous studies have demonstrated that particles with moderate shell thicknesses (2–3 monolayers) exhibit superior performance compared to those with thin monolayers.^[^ ^68^, ^69^ ^]^ Pd@Pt nanoparticles with a 0.94 nm Pt shell show improved specific activity due to the faster reduction of OH_ad_ and weaker bonding strengths compared to Pt/C.^[^ ^60^ ^]^ A previous study has shown that the ORR activity of Pt_1.2nm_/Pd(111) with a Pt shell thickness of ≈3 monolayers is four times higher than that of Pt(111), and its durability is also enhanced due to the compressive surface strains induced by the mismatch between the Pt(111) and Pd(111) lattices.^[^ ^70^ ^]^ A study using density functional theory calculations on Pd@Pt/C catalysts with one, two, and three monolayer Pt shells demonstrates that the enhancement of specific activity is primarily driven by compressive strain effects. This also reveals the facet‐dependent influence of the nanosize‐induced surface contraction on the oxygen‐binding energy.^[^ ^71^ ^]^ The improved catalytic activity is primarily because Pd modifies the Pt 5*d* electronic structure, accelerating the hydrogenation of O, weakening the Pt–OH adsorption, and enhancing ORR kinetics.^[^ ^72^ ^]^ A previous study also indicates that electron transfer from Pd to Pt reduces the activation energy of the rate‐limiting step of the ORR, thereby increasing the atomic efficiency of Pt.^[^ ^67^ ^]^

The CV curves in Figure 6 show that the potential of the Pt oxide reduction peak for the connected Pd@Pt_0.8_ catalyst is higher than those for the Pd@Pt_0.3_, Pd@Pt_1.5_ and Pt/C catalysts, suggesting its weaker Pt–OH adsorption. TEM analysis shows that the Pd@Pt_0.8_ catalyst has a lattice mismatch of 2.5%, leading to compressive strain, which likely contributes to its increased activity. STEM–EDX line mapping reveals a Pt shell thickness of 1.1 nm, which corresponds to ≈3.5 monolayers. The XPS results show a significant downshift in the Pt 4*f* binding energies for the Pd@Pt_0.8_ catalyst, indicating a modified electronic structure with a higher electron cloud density around the Pt atoms, which enhances the ORR activity.^[^ ^73^, ^74^, ^75^ ^]^ Furthermore, using the Koutecky–Levich plots (Figure S1, Supporting Information), the electron transfer number for ORR on the connected Pd@Pt_0.8_ catalyst was estimated to be ≈4 (*n* = 3.95) at 0.4, 0.85, and 0.9 V vs. reversible hydrogen electrode (RHE). The reduction of oxygen molecules to water via a four‐electron transfer is desirable for efficient energy conversion.^[^ ^76^ ^]^ This result suggests that the developed Pd@Pt_0.8_ catalyst facilitates the oxygen reduction process. The EIS plots in Figure S2 (Supporting Information) show that at 0.9 V, the Pd@Pt_0.8_ catalyst exhibits a smaller semicircle than Pt/C, corresponding to a lower charge transfer resistance (Rct).^[^ ^77^ ^]^ The favorable reaction kinetics and rapid charge transfer confirm the excellent electrocatalytic performance of Pd@Pt_0.8_, consistent with the CV and LSV data.

Furthermore, a connected nanoparticle structure can also contribute to improved specific activity. Previous studies have emphasized the effects of Pt coordination numbers on electrocatalytic activity. Surfaces with concave defects can exceed the activity of Pt(111), as convex defects found in regular nanoparticles are less active. A concave structure with higher coordination numbers leads to optimal adsorption energies of ORR intermediates like *OH, thus boosting specific activity.^[^ ^78^ ^]^ Another study shows that proper Pt coordination site exposure in grain boundaries of porous Pt provides optimal adsorption energies for oxygen species, leading to enhanced ORR activity, even surpassing Pt(111) surfaces.^[^ ^79^ ^]^ As shown in the TEM image in Figure 3, the concave structure of the connected Pd@Pt_0.8_ nanoparticles would have enhanced the ORR surface‐specific activity.

These results suggest the structural advantages of the connected core–shell design. Further comprehensive structural analyses are required to fully understand the reasons underlying the activity improvements.

### 2.3 Load Cycle Durability of the Connected Pd@Pt Catalysts

**Figure 7:**
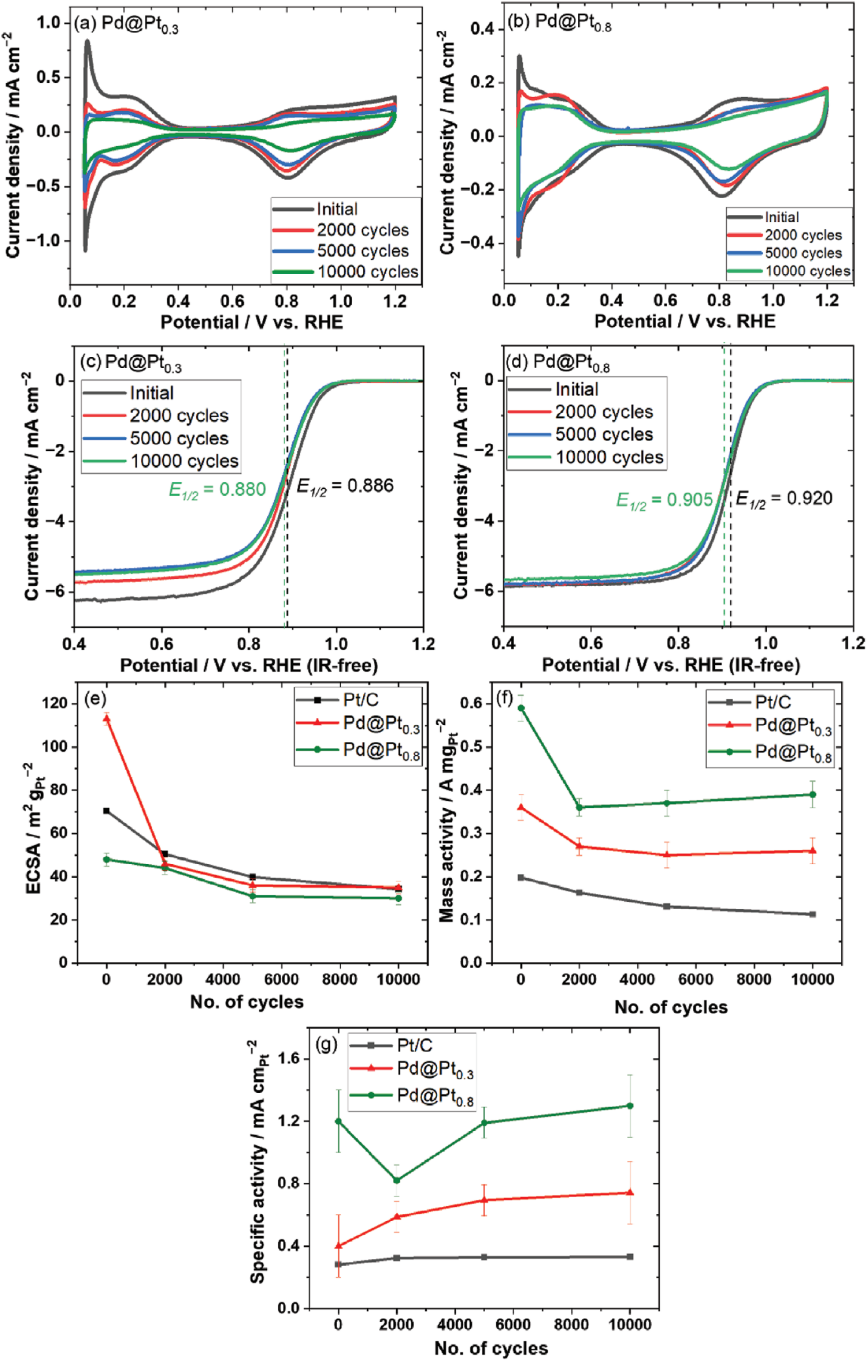
Load cycle durability test in a 0.1 m HClO_4_ aqueous solution at 60 °C. CV (N_2_ saturated atmosphere, scan rate: 20 mV s^−1^) of a) Pd@Pt_0.3_ and b) Pd@Pt_0.8_ catalysts after different durability cycles. LSV curves (O_2_ saturated atmosphere, scan rate: 20 mV s^−1^, scan speed: 1600 rpm) of c) Pd@Pt_0.3_ and d) Pd@Pt_0.8_ catalysts after different durability cycles. e) ECSA f) mass activity, and g) specific activity of the Pd@Pt_0.3_ and Pd@Pt_0.8_ catalysts and the commercial Pt/C catalyst before and after a specific number of load cycles.

Despite their potential for enhancing the ORR, the stability and durability of Pd@Pt core–shell catalysts remain a critical concern. The lower redox potential of the Pd core (0.95 V vs. SHE) compared to that of Pt (1.18 V vs. SHE) makes it more susceptible to dissolution under cathode conditions involving high potential, low pH, and elevated temperature. Previous studies have observed the dissolution of the Pd core during single‐cell operation using a carbon‐supported Pd core‐Pt shell‐structured catalyst (Pt/Pd/C), where the formation of a Pd band in the Nafion^®^ membrane is detected after a potential cycling durability test.^[^ ^61^, ^80^, ^81^ ^]^ Hence, to confirm the durability of the connected core–shell Pd@Pt catalysts, an accelerated durability test (ADT) was conducted using the Pd@Pt_0.3_ and Pd@Pt_0.8_ catalysts because of their better ORR performance among the synthesized catalysts. The test involved load cycles (0.6 V for 3 s and 1.0 V for 3 s), as shown in Figure S3 (Supporting Information), in an N_2_‐saturated 0.1 M HClO_4_ solution at 60 °C for 10,000 cycles. Additional details regarding the load cycle durability test are provided in the Experimental section of Supplementary Information. The CV and LSV curves of the connected Pd@Pt_0.3_ and Pd@Pt_0.8_ catalysts were recorded after 0, 2000, 5000, and 10,000 load cycles (**Figure** **7**a–d). The CV curves after ADT reveal differences in the hydrogen adsorption/desorption peaks during cycling. For both catalysts, the sharp hydrogen adsorption/desorption of Pd in the 0.05–0.1 V region diminish gradually after 2000 cycles and vanish after 5000 cycles. In addition, the oxide reduction peaks shift toward the higher potentials. The observed changes in the Pd hydrogen adsorption/desorption peaks and the oxide reduction peaks are significant for understanding the surface modifications of the catalysts during repeated load cycles.^[^ ^80^, ^82^ ^]^ The LSV curves show an initial decrease in the ORR activity, which is stabilized after 2000 cycles with a slight increase after ≈10,000 cycles. After ADT, the connected Pd@Pt_0.3_ and Pd@Pt_0.8_ catalysts exhibit minor shifts in *E₁_/_₂* (6 and 15 mV, respectively).

The ECSA, mass activity, and specific activity variations of the connected Pd@Pt_0.3_, Pd@Pt_0.8_, and commercial Pt/C catalysts are shown in Figure 7e–g, respectively. The Pt/C catalyst experiences an ECSA loss of ≈43% after 5000 cycles and a continuous loss of ≈51% after 10,000 cycles of ADT. This decrease is a collective result of nanoparticle growth via Pt dissolution and Oswald ripening, nanoparticle migration and coalescence on the carbon support, and detachment of Pt nanoparticles from the carbon support.^[^ ^4^ ^]^ Conversely, the Pd@Pt_0.3_ catalyst with more exposed Pd particles exhibits a greater ECSA loss (≈60%) after the first 5000 cycles, which tends to stabilize afterward. The Pd@Pt_0.8_ catalyst, with comparatively less exposed Pd on the surface, experiences a smaller initial ECSA loss (≈37%) after 5000 cycles, maintaining stability throughout the subsequent 10,000 cycles of ADT. During the durability test, the repeated load cycles often lead to alterations in the surface structure, as highlighted in numerous studies.^[^ ^80^, ^82^ ^]^ The CV curves indicate potential smoothing of the irregularly deposited Pt shell and an increase in the Pt shell coverage on the catalyst surface. Additionally, the ECSA loss of the Pd@Pt_0.8_ catalyst is likely attributed to the reduced surface roughness owing to surface Pt reorganization.^[^ ^83^ ^]^

The mass activities of the Pd@Pt_0.3_ and Pd@Pt_0.8_ catalysts initially decrease and remain almost constant after 2000 cycles. However, the specific activity increases after 2000 cycles, surpassing the initial value after 10,000 cycles. This trend is consistent for both the Pd@Pt_0.3_ and Pd@Pt_0.8_ catalysts, suggesting that the catalyst surfaces were rearranged to enhanced ORR activity during load cycles. This activity enhancement results from the rearrangement of the surface Pt atoms during ADT, which decreases the number of low‐coordinated Pt atoms. A similar study has demonstrated that an initially non‐uniform Pt shell containing numerous defects and low‐coordinated Pt atoms undergoes surface diffusion during ADT.^[^ ^84^ ^]^ This process allows Pt atoms to migrate to more stable sites, reducing the number of low‐coordinated atoms and enhancing the catalytic activity. During ADT, the rearrangement of Pt atoms on the connected Pd@Pt catalysts optimizes the surface structure of the Pt shell, stabilizes the ECSA, and increases the specific activity.

Table S2 (Supporting Information) provides a comprehensive comparison of the electrochemical performance and load cycle durability of recently reported carbon‐support‐free catalysts. The catalyst developed in this study exhibits relatively high ORR activity and surface‐specific activity retention against load cycling at high temperatures and in an acidic electrolyte solution.

**Figure 8:**
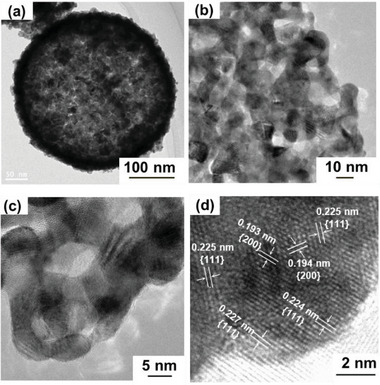
a–d) TEM images of the connected Pd@Pt_0.8_ catalyst after 10,000 load cycles of ADT.

After 10,000 load cycles, the structural durability of the connected Pd@Pt_0.8_ catalyst was assessed by TEM images. As shown in **Figure** **8**a–c, the nanonetwork and porous hollow capsule structure are maintained, and the average crystallite size is 10 ± 2.0 nm. Figure 8d shows the lattice fringes after the ADT, wherein the measured lattice spacings of 0.193 and 0.194 nm correspond to the {200} planes of Pt, and those of 0.224, 0.225, and 0.227 nm correspond to the {111} planes. After ADT, the average lattice spacings of the connected Pd@Pt_0.8_ catalyst change slightly from 0.191 to 0.194 nm for the {200} plane and from 0.221 to 0.225 nm for the {111} plane, indicating surface reconstruction and reorientation of the Pt atoms during potential cycling, which helped to explain the CV curves.

**Figure 9:**
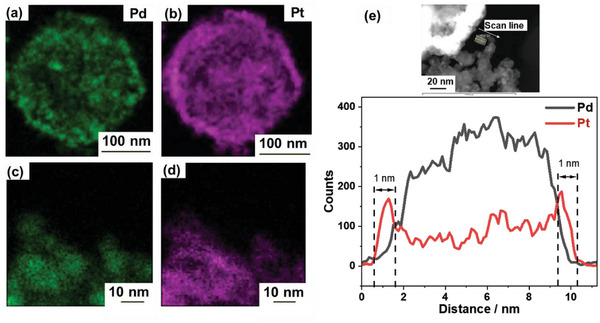
a–d) STEM–EDX elemental mapping and e) STEM–EDX line scan along the arrow of the connected Pd@Pt_0.8_ catalyst after 10,000 load cycles of ADT.

STEM–EDX area mapping and line mapping images (**Figure** **9**c,d) of the connected Pd@Pt_0.8_ catalyst after 10,000 ADT cycles further confirm their structural integrity. The images reveal that the core–shell structure remains intact with a uniform distribution of Pd and Pt. The EDX analysis shows that the compositions of the connected Pd@Pt_0.8_ catalyst (atomic %) before and after ADT are Pd_58_Pt_41_ (Pt/Pd = 0.7) and Pd_55_Pt_44_ (Pt/Pd = 0.8), respectively, suggesting minimal dissolution of the Pd core by Ostwald ripening. This stability is likely due to the relatively large size of the synthesized Pd nanoparticles (≈8 nm). Studies have indicated that larger particles (>6 nm) exhibit decreased dissolution compared to smaller particles. The oxophilic feature can lead to the formation of a dissolution‐inhibiting passivation layer of PtO*_x_*.^[^ ^85^ ^]^ This is corroborated by the STEM–EDX line mapping images, as the intensities of the Pt and Pd peaks remain almost unchanged even after 10,000 load cycles (Figures 4e and 9e). The Pt shell thickness is 1.0 nm for the Pd@Pt_0.8_ catalyst after ADT with a uniform Pt coverage, which corresponds to ≈3.5 monolayers of Pt.

The above results suggest a significant rearrangement of the Pt atoms on the surface, which results in the formation of a more uniform Pt shell layer with enhanced durability during load cycling (as illustrated in Scheme S1, Supporting Information). The initial phase of the load cycle test, as evident from the CV curves, indicates a surface structural change, confirming the development of a more uniform Pt shell layer. After 5000 cycles, neither the ECSA nor the ORR activity decreases, highlighting the remarkable durability of the more uniform Pt shell layer against load cycles. The developed carbon‐support‐free catalyst exhibits exceptional durability against carbon corrosion, especially during start‐up and shut‐down cycles. Furthermore, as shown in Figure S4 (Supporting Information), the connected Pd@Pt_0.8_ catalyst also demonstrates higher methanol and CO tolerances compared to Pt/C, underscoring its potential application in other fuel cell systems.

## 3 Conclusion

This study demonstrates an innovative low‐temperature Pt atomic shell deposition method for synthesizing support‐free connected Pd@Pt catalysts. This approach effectively tackles several challenges encountered in previously developed connected catalysts, including undesirable grain growth, low surface area, and degradation associated with the presence of Fe. The resulting connected Pd@Pt_0.8_ catalyst shows high ORR activity and load cycle durability because of the optimal Pt shell thickness, improved surface properties, and unique connected core–shell structure. The catalyst retains ≈70% of its mass activity and 100% of its specific activity after 10,000 load cycles, thereby effectively addressing the carbon corrosion issues of conventional carbon‐supported Pt‐based nanoparticle catalysts while delivering enhanced ORR activity and load cycle durability. This versatile method can be extended to other inexpensive nonprecious metals, which will be the focus of our future research.

This study established a foundational understanding of the synthesis, structure, and electrochemical properties of core‐shell type connected nanoparticle catalyst materials. This newly developed catalyst possesses a porous hollow capsule structure, which is same as that of connected Pt–Fe alloy catalyst showing practical MEA performance in our previous report.^[^ ^86^ ^]^ Compared with conventional Pt–Fe alloy catalysts, a higher surface area of the developed catalyst offers advantages for MEA performance, however, MEA performance depends on various complex parameters beyond the catalyst itself. Therefore, a thorough assessment including structural characterization and electrochemical analysis for MEAs will be required to fully leverage the benefits of increased surface area. This is a central focus of our future work, which can provide new scientific insights on the design of carbon‐free catalyst layers with connected nanoparticle catalysts. Furthermore, ongoing enhancements to this system and the demonstration of improved durability under actual fuel cell operating conditions hold promise for advancing fuel cell technology. This study represents a crucial step toward the development of more efficient and durable support‐free catalysts, contributing to the progress of clean energy technologies.

## Conflict of Interest

The authors declare no conflict of interest.

## Supporting information



Supporting Information

## Data Availability

The data that support the findings of this study are available from the corresponding author upon reasonable request.

## References

[1] I. Staffell , D. Scamman , A. Velazquez Abad , P. Balcombe , P. E. Dodds , P. Ekins , N. Shah , K. R. Ward , Energy Environ. Sci. 2019, 12, 463.

[2] F. Zhu , L. Luo , A. Wu , C. Wang , X. Cheng , S. Shen , C. Ke , H. Yang , J. Zhang , ACS Appl. Mater. Interfaces 2020, 12, 26076. 32412233 10.1021/acsami.0c06981

[3] B. G. Pollet , S. S. Kocha , I. Staffell , Curr. Opin. Electrochem. 2019, 16, 90.

[4] J. Zhao , Z. Tu , S. H. Chan , J. Power Sources 2021, 488, 229434.

[5] Y. Wang , D. Wang , Y. Li , SmartMat 2021, 2, 56.

[6] M. K. Debe , Nature 2012, 486, 43. 22678278 10.1038/nature11115

[7] I. E. L. Stephens , A. S. Bondarenko , F. J. Perez‐Alonso , F. Calle‐Vallejo , L. Bech , T. P. Johansson , A. K. Jepsen , R. Frydendal , B. P. Knudsen , J. Rossmeisl , I. Chorkendorff , J. Am. Chem. Soc. 2011, 133, 5485. 21417329 10.1021/ja111690g

[8] V. Stamenkovic , B. S. Mun , K. J. J. Mayrhofer , P. N. Ross , N. M. Markovic , J. Rossmeisl , J. Greeley , J. K. Nørskov , Angew. Chem. Int. Ed Engl. 2006, 45, 2897. 16596688 10.1002/anie.200504386

[9] S. Kim , Y. Kang , H. C. Ham , Energies 2021, 14, 7814.

[10] M. Liu , Z. Zhao , X. Duan , Y. Huang , Adv. Mater. 2019, 31, 1802234. 10.1002/adma.201802234 30561854

[11] J. Greeley , I. E. L. Stephens , A. S. Bondarenko , T. P. Johansson , H. A. Hansen , T. F. Jaramillo , J. Rossmeisl , I. Chorkendorff , J. K. Nørskov , Nat. Chem. 2009, 1, 552. 21378936 10.1038/nchem.367

[12] X. Huang , Z. Zhao , L. Cao , Y. Chen , E. Zhu , Z. Lin , M. Li , A. Yan , A. Zettl , Y. M. Wang , X. Duan , T. Mueller , Y. Huang , Science 2015, 348, 1230. 26068847 10.1126/science.aaa8765

[13] Y. Yao , Q. Dong , A. Brozena , J. Luo , J. Miao , M. Chi , C. Wang , I. G. Kevrekidis , Z. J. Ren , J. Greeley , G. Wang , A. Anapolsky , L. Hu , Science 2022, 376, eabn3103. 35389801 10.1126/science.abn3103

[14] X. Han , G. Wu , S. Zhao , J. Guo , M. Yan , X. Hong , D. Wang , Matter 2023, 6, 1717.

[15] W. Yan , W. Chen , Y. Chen , Adv. Funct. Mater. 2024, 34, 2401027.

[16] Y. Kang , O. Cretu , J. Kikkawa , K. Kimoto , H. Nara , A. S. Nugraha , H. Kawamoto , M. Eguchi , T. Liao , Z. Sun , T. Asahi , Y. Yamauchi , Nat. Commun. 2023, 14, 4182. 37443103 10.1038/s41467-023-39157-2 PMC10344865

[17] Z. Wang , S. Chen , W. Wu , R. Chen , Y. Zhu , H. Jiang , L. Yu , N. Cheng , Adv. Mater. 2023, 35, 2301310. 10.1002/adma.202301310 37196181

[18] J. Li , S. Sharma , X. Liu , Y.‐T. Pan , J. S. Spendelow , M. Chi , Y. Jia , P. Zhang , D. A. Cullen , Z. Xi , H. Lin , Z. Yin , B. Shen , M. Muzzio , C. Yu , Y. S. Kim , A. A. Peterson , K. L. More , H. Zhu , S. Sun , Joule 2019, 3, 124.

[19] T. Ingsel , R. Gupta , in Nanomaterials for Electrocatalysis (Eds: T. Maiyalagan , M.. Khandelwal , A.. Kumar , T. A.. Nguyen , G. Yasin ), Elseivier, Amsterdam, Netherlands 2022, Ch. 5.

[20] L. Du , V. Prabhakaran , X. Xie , S. Park , Y. Wang , Y. Shao , Adv. Mater. 2021, 33, 1908232. 10.1002/adma.201908232 32240570

[21] L. Osmieri , Q. Meyer , Curr. Opin. Electrochem. 2022, 31, 100847.

[22] L. Chong , J. Wen , J. Kubal , F. G. Sen , J. Zou , J. Greeley , M. Chan , H. Barkholtz , W. Ding , D.‐J. Liu , Science 2018, 362, 1276. 30409809 10.1126/science.aau0630

[23] M. Dan , X. Zhang , Y. Yang , J. Yang , F. Wu , S. Zhao , Z.‐Q. Liu , Proc. Natl. Acad. Sci. 2024, 121, e2318174121. 38289955 10.1073/pnas.2318174121 PMC10861853

[24] H. Wang , T. Yang , J. Wang , Z. Zhou , Z. Pei , S. Zhao , Chem 2024, 10, 48.

[25] T. Kwon , M. Jun , K. Lee , Adv. Mater. 2020, 32, 2001345. 10.1002/adma.202001345 32633878

[26] H. Y. Kim , T. Kwon , Y. Ha , M. Jun , H. Baik , H. Y. Jeong , H. Kim , K. Lee , S. H. Joo , Nano Lett. 2020, 20, 7413. 32924501 10.1021/acs.nanolett.0c02812

[27] Z. Guo , Z. Zhang , Z. Li , M. Dou , F. Wang , Nano Energy 2019, 57, 108.

[28] Z. Yao , Y. Yuan , T. Cheng , L. Gao , T. Sun , Y. Lu , Y.‐G. Zhou , P. L. Galindo , Z. Yang , L. Xu , H. Yang , H. Huang , Nano Lett. 2021, 21, 9354. 34719926 10.1021/acs.nanolett.1c03805

[29] M. Li , Z. Zhao , T. Cheng , A. Fortunelli , C.‐Y. Chen , R. Yu , Q. Zhang , L. Gu , B. V. Merinov , Z. Lin , E. Zhu , T. Yu , Q. Jia , J. Guo , L. Zhang , W. A. Goddard , Y. Huang , X. Duan , Science 2016, 354, 1414. 27856847 10.1126/science.aaf9050

[30] H. Xu , H. Shang , C. Wang , Y. Du , Adv. Funct. Mater. 2020, 30, 2000793.

[31] H. Jin , Z. Xu , Z.‐Y. Hu , Z. Yin , Z. Wang , Z. Deng , P. Wei , S. Feng , S. Dong , J. Liu , S. Luo , Z. Qiu , L. Zhou , L. Mai , B.‐L. Su , D. Zhao , Y. Liu , Nat. Commun. 2023, 14, 1518. 36934107 10.1038/s41467-023-37268-4 PMC10024750

[32] C. Wang , Y. Ha , X. Mao , W. Xu , A. Du , R. Wu , S. Chou , H. Zhang , Adv. Mater. Interfaces 2022, 9, 2101877.

[33] S. Ajmal , A. Kumar , G. Yasin , M. M. Alam , M. Selvaraj , M. Tabish , M. A. Mushtaq , R. K. Gupta , W. Zhao , J. Alloys Compd. 2023, 943, 169067.

[34] Q. Chen , Z. Chen , A. Ali , Y. Luo , H. Feng , Y. Luo , P. Tsiakaras , P. K. Shen , Chem. Eng. J. 2022, 427, 131565.

[35] Z. Lyu , J. Cai , X. Zhang , H. Li , H. Huang , S. Wang , T. Li , Q. Wang , Z. Xie , S. Xie , Adv. Mater. 2024, 36, 2314252. 10.1002/adma.202314252 38551140

[36] L. Zhang , L. T. Roling , X. Wang , M. Vara , M. Chi , J. Liu , S.‐I. Choi , J. Park , J. A. Herron , Z. Xie , M. Mavrikakis , Y. Xia , Science 2015, 349, 412. 26206931 10.1126/science.aab0801

[37] G. Montserrat‐Sisó , B. Wickman , Electrochim. Acta 2022, 420, 140425.

[38] J. D. Sinniah , W. Y. Wong , K. S. Loh , R. M. Yunus , S. N. Timmiati , J. Power Sources 2022, 534, 231422.

[39] G. W. Sievers , A. W. Jensen , J. Quinson , A. Zana , F. Bizzotto , M. Oezaslan , A. Dworzak , J. J. K. Kirkensgaard , T. E. L. Smitshuysen , S. Kadkhodazadeh , M. Juelsholt , K. M. Ø. Jensen , K. Anklam , H. Wan , J. Schäfer , K. Čépe , M. Escudero‐Escribano , J. Rossmeisl , A. Quade , V. Brüser , M. Arenz , Nat. Mater. 2021, 20, 208. 32839587 10.1038/s41563-020-0775-8

[40] L. Peles‐Strahl , Y. Persky , L. Elbaz , SusMat 2023, 3, 44.

[41] S. Kumar , A. Yoyakki , A. Pandikassala , R. Soni , S. Kurungot , Adv. Sustain. Syst. 2023, 7, 2200330.

[42] Q. Sun , X.‐H. Li , K.‐X. Wang , T.‐N. Ye , J.‐S. Chen , Energy Environ. Sci. 2023, 16, 1838.

[43] T. Tamaki , H. Kuroki , S. Ogura , T. Fuchigami , Y. Kitamoto , T. Yamaguchi , Energy Environ. Sci. 2015, 8, 3545.

[44] H. Kuroki , M. Matsumoto , T. Tamaki , H. Imai , T. Yamaguchi , J. Chem. Eng. Jpn. 2023, 56, 2197946.

[45] H. Kuroki , T. Tamaki , T. Yamaguchi , J. Electrochem. Soc. 2016, 163, F927.

[46] H. Kuroki , Y. Imura , R. Fujita , T. Tamaki , ACS Appl. Nano Mater. 2020, 3, 9912.

[47] J. An , N. Li , Y. Wu , S. Wang , C. Liao , Q. Zhao , L. Zhou , T. Li , X. Wang , Y. Feng , Environ. Sci. Technol. 2020, 54, 10916. 32786563 10.1021/acs.est.0c03233

[48] H. Inoue , E. Higuchi , Core‐Shell Yolk‐Shell Nanocatalysts, (Eds: H. Yamashita , H. Li ), Springer, Singapore, 2021, pp. 275–288.

[49] Y. Cong , H. Wang , F. Meng , D. Dou , X. Meng , Q. Zhao , D. Cao , Y. Wang , J. Solid State Electrochem. 2022, 26, 1381.

[50] K. Sasaki , K. A. Kuttiyiel , R. R. Adzic , Curr. Opin. Electrochem. 2020, 21, 368.

[51] X. Zhao , K. Sasaki , Acc. Chem. Res. 2022, 55, 1226. 35451817 10.1021/acs.accounts.2c00057

[52] C. Liu , T. Uchiyama , K. Yamamoto , T. Watanabe , X. Gao , H. Imai , M. Matsumoto , S. Sugawara , K. Shinohara , K. Oshima , S. Sakurai , Y. Uchimoto , ACS Appl. Energy Mater. 2021, 4, 810.

[53] A. S. Nair , B. Pathak , J. Phys. Chem. C 2019, 123, 3634.

[54] S.‐I. Choi , M. Shao , N. Lu , A. Ruditskiy , H.‐C. Peng , J. Park , S. Guerrero , J. Wang , M. J. Kim , Y. Xia , ACS Nano 2014, 8, 10363. 25247667 10.1021/nn5036894

[55] Y. Zhang , J. Qin , D. Leng , Q. Liu , X. Xu , B. Yang , F. Yin , J. Power Sources 2021, 485, 229340.

[56] S. Yamazaki , M. Asahi , N. Taguchi , T. Ioroi , Y. Kishimoto , H. Daimon , M. Inaba , K. Koga , Y. Kurose , H. Inoue , ACS Catal. 2020, 10, 14567.

[57] X. Li , Y. Liu , W. Bi , J. Bi , R. Guo , R. Li , C. Wang , Q. Zhan , W. Wang , S. Yang , F. Shi , J. Wu , M. Jin , J. Mater. Chem. A 2020, 8, 16477.

[58] H. Li , G. Li , J. Mater. Chem. A 2023, 11, 9383.

[59] Y. Hashiguchi , I. Nakamura , T. Honma , T. Matsushita , H. Murayama , M. Tokunaga , Y.‐K. Choe , T. Fujitani , ChemPhysChem 2023, 24, 202200389. 10.1002/cphc.202200389 36089540

[60] R. Choi , S.‐I. Choi , C. H. Choi , K. M. Nam , S. I. Woo , J. T. Park , S. W. Han , Chem. – Eur. J. 2013, 19, 8190. 23613263 10.1002/chem.201203834

[61] K. Sasaki , H. Naohara , Y. Choi , Y. Cai , W.‐F. Chen , P. Liu , R. R. Adzic , Nat. Commun. 2012, 3, 1115. 23047673 10.1038/ncomms2124

[62] X. Wang , M. Vara , M. Luo , H. Huang , A. Ruditskiy , J. Park , S. Bao , J. Liu , J. Howe , M. Chi , Z. Xie , Y. Xia , J. Am. Chem. Soc. 2015, 137, 15036. 26566188 10.1021/jacs.5b10059

[63] J. C. Fuggle , N. Mårtensson , J. Electron Spectrosc. Relat. Phenom. 1980, 21, 275.

[64] J. Yang , J. Y. Lee , Q. Zhang , W. Zhou , Z. Liu , J. Electrochem. Soc. 2008, 155, B776.

[65] W. Wang , Z. Wang , J. Wang , C.‐J. Zhong , C.‐J. Liu , Adv. Sci. 2017, 4, 1600486. 10.1002/advs.201600486 PMC5396164 28435780

[66] N. Aoki , H. Inoue , R. Yoshiura , Y. Hasegawa , S. Miyazaki , A. Suzuki , H. Daimon , T. Doi , M. Inaba , K. Higashi , T. Uruga , Y. Iwasawa , H. Tanida , Q. Yuan , N. Takao , H. Imai , T. Mikami , A. Daimaru , J. Electrochem. Soc. 2020, 167, 044513.

[67] Y. Hashiguchi , I. Nakamura , T. Honma , T. Matsushita , H. Murayama , M. Tokunaga , Y.‐K. Choe , T. Fujitani , Chemphyschem 2023, 24, e202200389. 36089540 10.1002/cphc.202200389

[68] L. Yang , M. B. Vukmirovic , D. Su , K. Sasaki , J. A. Herron , M. Mavrikakis , S. Liao , R. R. Adzic , J. Phys. Chem. C 2013, 117, 1748.

[69] G. M. Leteba , D. R. G. Mitchell , P. B. J. Levecque , E. van Steen , C. I. Lang , RSC Adv. 2020, 10, 29268. 35521089 10.1039/d0ra05195k PMC9055937

[70] Y. Bando , Y. Takahashi , E. Ueta , N. Todoroki , T. Wadayama , J. Electrochem. Soc. 2015, 162, F463.

[71] J. X. Wang , H. Inada , L. Wu , Y. Zhu , Y. Choi , P. Liu , W.‐P. Zhou , R. R. Adzic , J. Am. Chem. Soc. 2009, 131, 17298. 19899768 10.1021/ja9067645

[72] F. H. B. Lima , J. Zhang , M. H. Shao , K. Sasaki , M. B. Vukmirovic , E. A. Ticianelli , R. R. Adzic , J. Phys. Chem. C 2007, 111, 404.

[73] S.‐H. Kwon , S.‐G. Lee , S.‐B. Han , K.‐W. Park , Electrocatalysis 2020, 11, 497.

[74] J. Fan , K. Qi , L. Zhang , H. Zhang , S. Yu , X. Cui , ACS Appl. Mater. Interfaces 2017, 9, 18008. 28488861 10.1021/acsami.7b05290

[75] H.‐U. Park , A.‐H. Park , W. Shi , G.‐G. Park , Y.‐U. Kwon , Ultrason. Sonochem. 2019, 58, 104673. 31554145 10.1016/j.ultsonch.2019.104673

[76] S. Li , L. Shi , Y. Guo , J. Wang , D. Liu , S. Zhao , Chem. Sci. 2024, 15, 11188. 39055002 10.1039/d4sc02853h PMC11268513

[77] R. Singh , D. Ruttala , T. Kar , A. Chakraborty , M. Neergat , J. Electrochem. Soc. 2015, 162, 489.

[78] F. Calle‐Vallejo , M. D. Pohl , D. Reinisch , D. Loffreda , P. Sautet , A. S. Bandarenka , Chem. Sci. 2017, 8, 2283. 28451330 10.1039/c6sc04788b PMC5363395

[79] H. Cheng , S. Liu , Z. Hao , J. Wang , B. Liu , G. Liu , X. Wu , W. Chu , C. Wu , Y. Xie , Chem. Sci. 2019, 10, 5589. 31293743 10.1039/c9sc01078e PMC6552488

[80] N. Aoki , H. Inoue , T. Okawa , Y. Ikehata , A. Shirai , H. Daimon , T. Doi , Y. Orikasa , Y. Uchimoto , H. Jinnai , S. Inamoto , Y. Otsuka , M. Inaba , Electrocatalysis 2018, 9, 125.

[81] K. Sasaki , H. Naohara , Y. Cai , Y. M. Choi , P. Liu , M. B. Vukmirovic , J. X. Wang , R. R. Adzic , Angew. Chem., Int. Ed. 2010, 49, 8602. 10.1002/anie.201004287 20931587

[82] D. Choi , J. Y. Jung , M. J. Lee , S. Kim , S. Lee , D. W. Lee , D. Kim , N. D. Kim , K.‐S. Lee , P. Kim , S. J. Yoo , ACS Catal. 2021, 11, 15098.

[83] M. F. Labata , G. Li , J. Ocon , P.‐Y. A. Chuang , J. Power Sources 2021, 487, 229356.

[84] M. Inaba , H. Daimon , J. Jpn. Pet. Inst. 2015, 58, 55.

[85] D. J. S. Sandbeck , N. M. Secher , F. D. Speck , J. E. Sørensen , J. Kibsgaard , I. Chorkendorff , S. Cherevko , ACS Catal. 2020, 10, 6281.

[86] B. Akbari , M. Pirhadi Tavandashti , M. Zandrahimi , Iran. J. Mater. Sci. Eng. 2011, 8, 48.

