# Supplementary material for: Support‐Free, Connected Core–Shell Nanoparticle Catalysts Synthesized via a Low‐Temperature Process for Advanced Oxygen Reduction Performance
Source: Adv Sci (Weinh). 2024 Dec 26;12(7):2408614. doi: 10.1002/advs.202408614 (PMC11831485; doi:10.1002/advs.202408614)
Supplement: Supplementary file 1 — Supporting Information [file ADVS-12-2408614-s001.docx]

**Supplementary Information**

**Support-free, Connected Core-shell Nanoparticle Catalysts Synthesized via a Low-Temperature Process for Advanced Oxygen Reduction Performance**

*Aparna Chitra Sudheer, ^a^ Gopinathan M. Anilkumar, ^a,b^ Hidenori Kuroki,^a^ Takeo Yamaguchi ^a^**

^a^ Laboratory for Chemistry and Life Sciences, Tokyo Institute of Technology, Yokohama, Kanagawa 226-8501, Japan

^b^ R&D Center, Noritake Co., Ltd., 300 Higashiyama, Miyoshi-cho, Aichi, 470-0293, Japan

*Corresponding author, email: yamag@res.titech.ac.jp

1. **Experimental section**
   1. **Synthesis of Pd_core_@Pt_shell_ (Pd@Pt) core-shell catalysts**

Initially, palladium (II) acetylacetonate (Pd(C_5_H_7_O_2_)_2_, Sigma‒Aldrich) was modified with polyvinyl-2-pyrrolidone (PVP, average *M*_w_: 55,000 g mol^−1^, Sigma‒Aldrich) in tetraethylene glycol (TEG, Sigma‒Aldrich). In a typical experiment, Pd(C_5_H_7_O_2_)_2_ (490 mg) and PVP (1.79 g) (PVP monomer unit/Pd = 10 mol/mol) were dispersed in TEG (70 mL). Subsequently, silica spheres with an average diameter of 320 nm (KE-P30, Nippon Shokubai Co., Ltd.) were coated with poly(diallyldimethylammonium chloride) (PDDA, Sigma-Aldrich) in a manner similar to that used for preparing the connected Pt‒Fe nanoparticle catalysts.^[1,2]^ A SiO_2_/PDDA (80 mg) solution in TEG (30 mL) was added to the Pd precursor dispersion and stirred overnight. Palladium nanoparticles were deposited on the surface-modified silica template by a polyol reaction at 100 °C for 3 h in a TEG medium under an Ar gas flow.

A Pt-shell precursor solution was prepared by dispersing 157.5 mg of potassium tetrachloroplatinate (K_2_PtCl_4_, Fujifilm Wako Pure Chemical Corporation) in TEG (10 mL). Subsequently, a Pt shell was developed on the Pd nanoparticles on the silica template (Pd/SiO_2_) by a polyol reaction with K_2_PtCl_4_ dispersed in TEG under an Ar gas flow. To achieve uniform Pt shell formation on the Pd nanoparticles and avoid forming Pt nanoparticles, a Pt precursor solution was added dropwise to the reaction solution. The shell structures were controlled by adjusting the temperature (80–100 °C), reaction time (3–20 h), and injection rate (0.02–1 mL min^−1^) during the shell formation reaction. A thin Pt shell (approximately a monolayer) was attained at 100 °C in 2 h at an injection rate of 1 mL min^−1^, while a moderate Pt shell (approximately 3 monolayers) was achieved at 100 °C in 3 h at an injection rate of 0.5 mL min^−1^. By reducing the injection rate to 0.02 mL min^−1^ and the temperature to 80 °C and extending the reaction time to 20 h, the Pt deposition rate decreased, and a catalyst with a thicker Pt shell (approximately six monolayers) was obtained while controlling the particle size. Catalysts with different Pt coverage were synthesized by varying the Pt/Pd ratio. After cooling, the samples were washed with ethanol and dried in an oven. Finally, core-shell connected Pd@Pt nanonetwork catalysts were obtained by dissolving the spherical silica template in a 3 M sodium hydroxide (NaOH (aq.), Fujifilm Wako Pure Chemical Corporation) solution at 80 °C, followed by washing with water and drying.

**1.2 Structural characterization**

To investigate the crystal structures, X-ray diffraction (XRD) measurements were carried out using an X-ray diffractometer (Ultima IV, Rigaku Corporation) operating at an accelerating voltage of 40 kV and a current of 40 mA using Cu Kα radiation (*λ* = 1.5406 Å). A Si non-reflective sample holder (Rigaku Corporation) was used, and the XRD data were collected over a 2*θ* range of 10−90° at a scan rate of 0.5° min^−1^. Reference XRD data for the peaks of fcc Pd and fcc Pt were obtained from the powder diffraction files available at the International Center for Diffraction Data (fcc Pd: PDF # 00-005-0681, fcc Pt: PDF # 00-004-0802). The crystallite sizes of the prepared catalysts were calculated using the Scherrer equation, which is expressed as *D* = *Kλ*/*β* cos *θ*, where *θ* is the Bragg angle, *β* is the full width at half maximum of the (111) peak, *λ* is the wavelength of the X-ray, and *K* is the shape factor (1.0747), assuming spherically shaped nanoparticles.^[3]^

The metal compositions of the catalysts were determined by inductively coupled plasma-atomic emission spectroscopy (ICP‒AES; Shimadzu ICPS-8100). From the ICP-AES data, the approximate thickness of the Pt atomic layers was calculated by assuming a conformally uniform Pt shell.^[4]^ The general morphology of the Pd@Pt catalysts before and after silica removal was analyzed by scanning electron microscopy (SEM, S-4800, Hitachi High-Technologies Corporation), and the nanoscale structures of the catalysts were observed by transmission electron microscopy (TEM, H-8100, Hitachi High-Technologies Corporation) at an accelerating voltage of 200 kV. Additionally, the metal profiles of the connected Pd@Pt networks were analyzed using scanning transmission electron microscopy–energy dispersive X-ray spectroscopy (STEM–EDX, HD-2700, Hitachi High-Technologies Corporation). The thickness of the Pt shell was estimated from the STEM–EDX line scan images.

The electronic properties of the as-prepared catalysts were examined using an X-ray photoelectron spectrometer (XPS, Quantum 2000, ULVAC-PHI Inc.) fitted with a twin-anode X-ray source using Al‒Kα radiation (*hν* = 1486.58 eV).

**1.3 Electrochemical characterization**

Electrochemical measurements were conducted using a potentiostat (HZ-7000, Meiden Hokuto Corporation) and an electrode rotating system (HZ-500, Meiden Denko Corporation) with a reversible hydrogen electrode (RHE) and a Pt wire as the reference and counter electrodes, respectively. The rotating disk electrode (RDE) method was used to evaluate the ORR and load-cycle durability of the connected Pd@Pt catalysts. Typically, the Pd@Pt catalyst (Pt loading of 17.3 μg_Pt_ cm^−2^ for all catalysts) was mixed with 5 wt% Nafion solution (12.5 μL, Nafion 1100EW, Sigma‒Aldrich Co. LLC) and 25% isopropanol solution (IPA, Fujifilm Wako Pure Chemical Corporation) in water (6.25 mL) and dispersed by sonication in an ice bath for more than one hour to form a catalyst ink. Subsequently, 10 μL of the ink was applied to a glassy carbon disk electrode with a geometric area of 0.196 cm^2^. To stabilize the voltammograms, the catalyst electrodes were electrochemically pretreated by performing 50 cycles of cyclic voltammetry (CV) in the potential range of 0.05−1.2 V vs. RHE at a sweep rate of 50 mV s^−1^ in a N_2_-saturated 0.1 M HClO_4_ aqueous solution at room temperature.

The ECSA of the Pd@Pt catalysts was assessed by measuring the hydrogen desorption peaks in the CV curves of the electrochemically pretreated electrodes. Linear sweep voltammetry (LSV) measurements were performed in an O_2_-saturated 0.1 M HClO_4_ solution at room temperature with a rotation rate of 1600 rpm and a sweep rate of 20 mV s^−1^. The mass and specific ORR activities were determined by calculating the kinetic current at 0.9 V vs. RHE from the IR-corrected LSV curves and then dividing it by the mass and surface area of Pt on the catalyst electrodes. LSV measurements were also performed at various electrode rotation rates (100, 400, 900, 1600, and 2500 rpm) to evaluate ORR kinetics. The number of electrons transferred for ORR was calculated from the slopes of the Koutecky–Levich plots at 0.9, 0.85, and 0.4 V vs. RHE. The EIS measurements were performed in an O_2_-saturated 0.1 M HClO_4_ solution at room temperature, with a rotation rate of 1600 rpm and a potential of 0.9 V, over a frequency range of 100,000 to 0.01 Hz. The load cycle durability of the catalysts was assessed by performing square-wave potential cycling in an N_2_-saturated 0.1 M HClO_4_ solution at 60 °C based on the Fuel Cell Commercialization Conference of Japan (FCCJ) protocol.^[5,6]^ The test involved potential cycling between 0.6 V and 1.0 V for 3 s each. The ECSAs and ORR activities of the connected Pd@Pt catalysts were estimated after different load-cycle intervals. The stability of the catalyst structures was evaluated by TEM, STEM-EDX elemental mapping, and line-scan measurements after 10,000 load cycles. In addition, the CV curves were measured in a 0.1 M HClO₄ aqueous solution containing 0.1 M methanol to assess the methanol and CO tolerances of the catalysts. The CO tolerance was evaluated by analyzing the ratio of the peak current in the forward scan (I*_f_*) to that in the backward scan (I*_b_*), represented as I*_f_/*I*_b_*.

The above electrochemical tests were conducted under the same conditions using a commercial Pt/C catalyst (TEC10E50E, Tanaka Kikinzoku Kogyo K.K.) to obtain reference data.

Table S1. Structural properties of the Pd@Pt catalysts with different atomic ratios.

| Catalyst | Reaction temperature (°C) | Reaction time (h) | Injection rate (mL min^−1^) | Peak position (2*θ*) | Crystallite size (nm) |
| --- | --- | --- | --- | --- | --- |
| Pd@Pt_0.3_ | 100 | 2 | 1.0 | 39.98 | 8.5 ± 1.0 |
| Pd@Pt_0.8_ | 100 | 3 | 0.5 | 39.90 | 10.0 ± 1.0 |
| Pd@Pt_1.5_ | 80 | 20 | 0.02 | 39.62 | 14.0 ± 2.0 |

The electron transfer numbers 𝑛 for ORR were estimated using equations (1) and (2) from Koutecky–Levich (K–L) plots, which represent the inverse of the measured current (1/𝐼) as a function of the inverse of the square root of the angular rotation rate (1/ω^1/2^). The rotation rates (100, 400, 900, 1600, and 2500 rpm) were used to obtain background-corrected LSV curves. These plots allowed for the separation of kinetic and diffusion-limited currents in the overall measured current, providing insight into the number of electrons transferred during the ORR process.^[7,8]^

$\frac{1}{I}=\frac{1}{I_{k}}+\frac{1}{I_{lim}}=\frac{1}{I_{k}}+\frac{1}{{B\omega}^{1/2}}$ (1)

where $I$ is the measured current, $I_{k}$is the kinetic current, and $I_{lim}$is the diffusion-limited current. $B$is the Levich constant, which is related to the properties of the electrolyte, the electrode, and the number of electrons transferred (𝑛) during the ORR. The angular rotation rate ($\omega$) is defined as

$$\omega=2\pi\times\frac{rpm}{60}$$

where rpm is the electrode rotation rate (rpm). By plotting $\frac{1}{I}$ vs. $\frac{1}{\omega^{1/2}}$, the slope of the line is used to calculate the electron transfer number 𝑛, which provides insights into the selectivity and pathway of the ORR.

The Levich constant is given by

$B=0.62nFA_{geo}D_{O_{2}}^{2/3}v^{-1/6}C_{O_{2}}$ (2)

where $F$ is the Faraday constant (96485 C mol^−1^),$A_{geo}$ is the geometric area of the electrode (0.196 cm²),$D_{O_{2}}$ is the oxygen diffusivity (1.93 × 10^−5^ cm² s^−1^),$v$ is the kinetic viscosity of the electrolyte (1.009 × 10^−2^ cm² s^−1^), and $C_{O_{2}}$ is the oxygen concentration (1.26 × 10^−6^ mol cm^−3^).


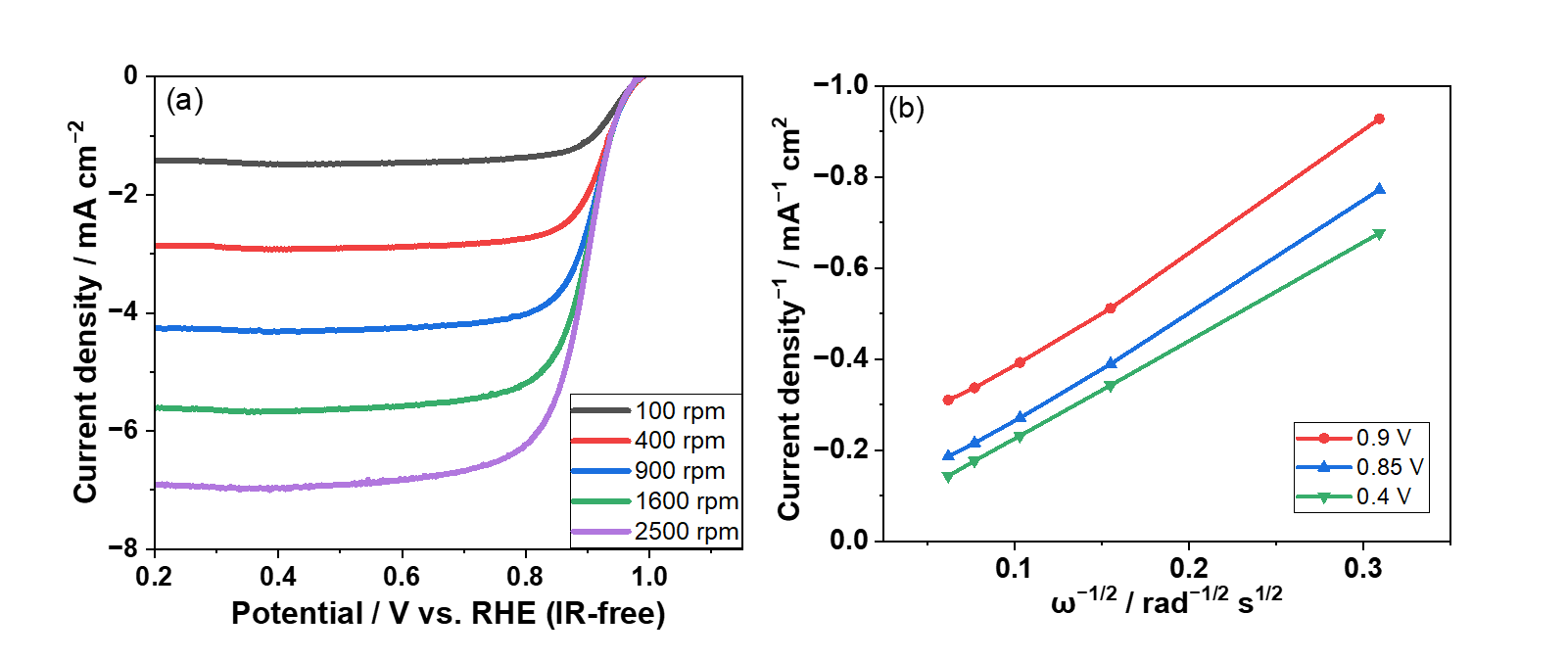


Figure S1. (a) LSV curves at different rotation rates and (b) K–L plots at different potentials of 0.9, 0.85, and 0.4 V vs. RHE for the connected Pd@Pt_0.8_ catalyst.


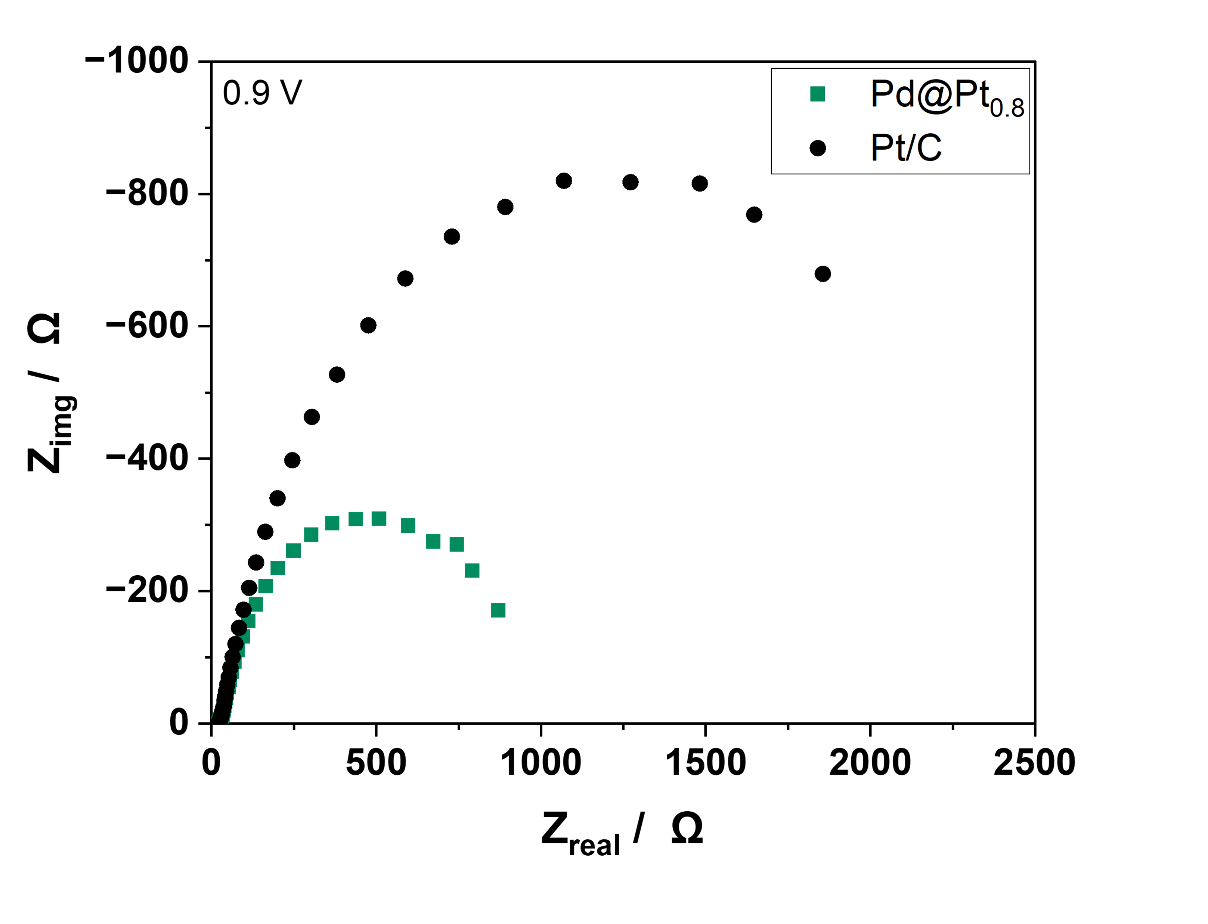


Figure S2. EIS plots of commercial Pt/C catalyst and the connected Pd@Pt_0.8_ catalyst in an O_2_-saturated 0.1 M HClO_4_ solution, with a rotation rate of 1600 rpm and a potential of 0.9 V.

Figure S3. Graphical representation of the potential sequence for ADT.


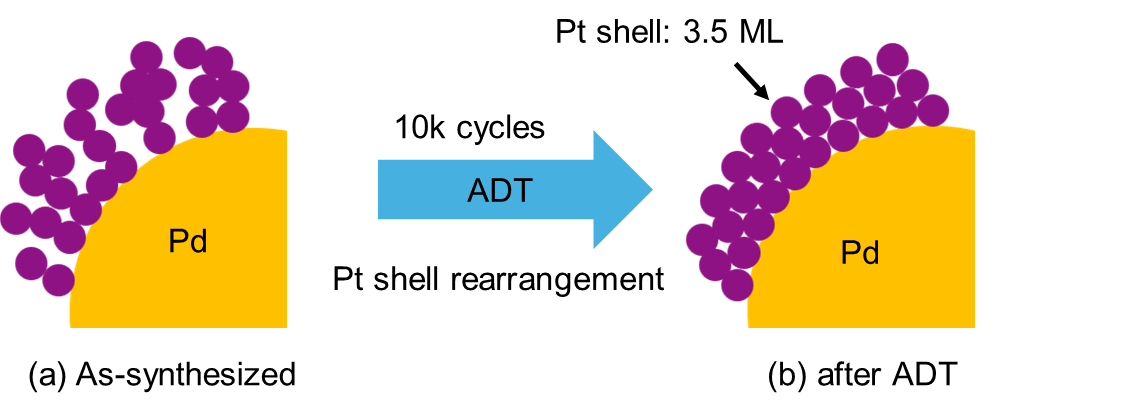


Scheme S1. Schematic of the surface structural modification during potential cycling on the support-free, connected Pd@Pt_0.8_ catalyst.

Figure S4 shows a comparison of the CV curves of the connected Pd@Pt_0.8_ catalyst and commercial Pt/C in an N_2_-saturated 0.1 M HClO_4_ in the presence of 0.1 M methanol. The current density of the methanol oxidation reaction (MOR) on the connected Pd@Pt_0.8_ catalyst was lower than that on Pt/C, indicating that the Pd@Pt_0.8_ catalyst was less active for the MOR compared to Pt/C. This finding aligns with a previous study, which showed that carbon-supported Pd–Pt nanoparticle catalysts were less active for the MOR in acidic media.^[9]^ A notable difference between the CV curves of these two catalysts is the peak current of the forward (I*_f_*) and backward (I*_b_*) scans. Because I*_b_* primarily reflects the oxidation of the residual CO poisoning species, a lower I*_f_*/I*_b_* value suggests better CO tolerance. The I*_f_*/I*_b_* ratio, an indicator of CO tolerance, was 0.70 for Pt/C—consistent with the results of previous studies^[10,11]^— and 1.11 for the connected Pd@Pt_0.8_ catalyst, confirming its higher CO tolerance.


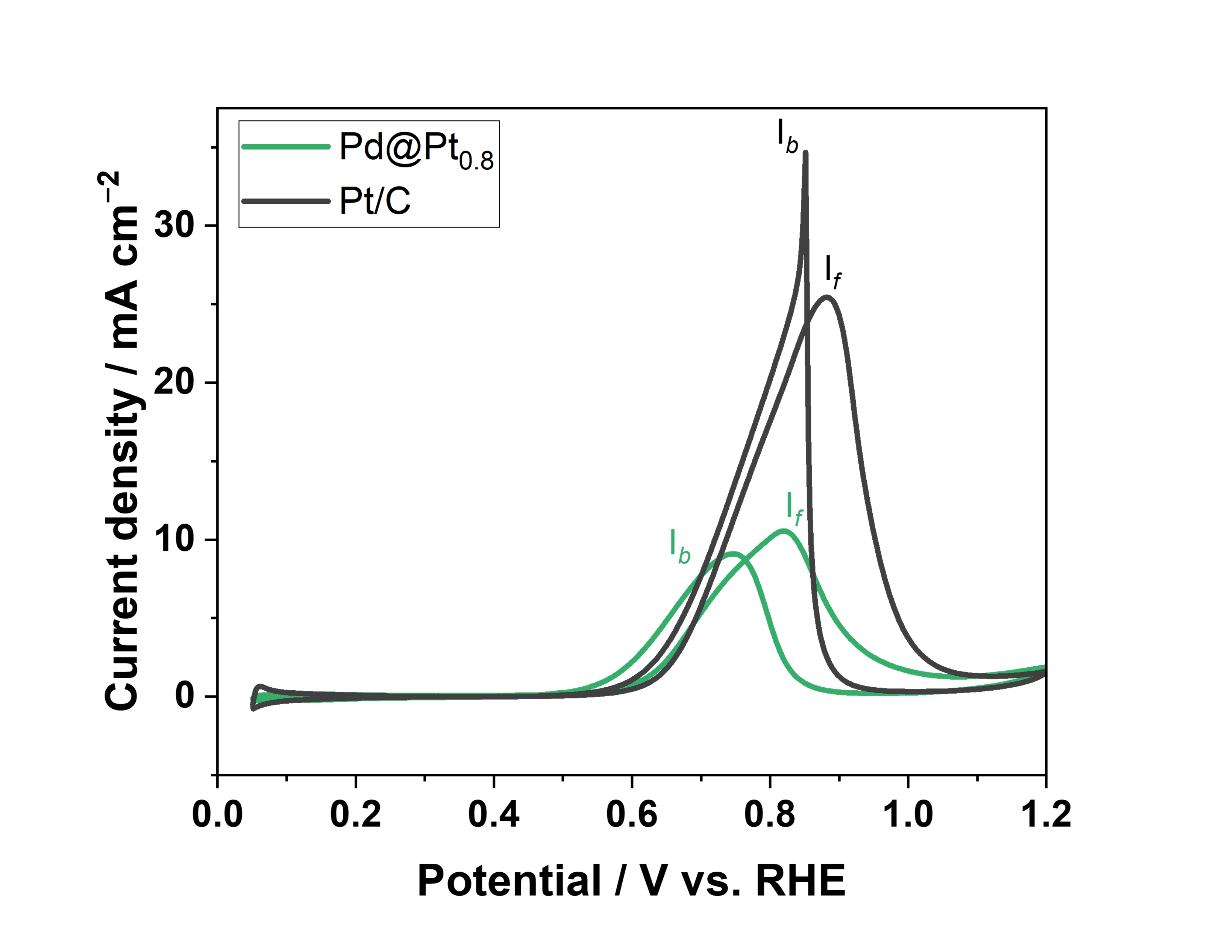


Figure S4. CV curves of a commercial Pt/C catalyst and the connected Pd@Pt_0.8_ catalyst in a N_2_-saturated 0.1 M HClO_4_ aqueous solution in the presence of 0.1 M methanol.

Table S2. Comparison of the ORR activities and durabilities for carbon-support-free catalysts reported in recent studies using RDE measurements (@0.9 V vs. RHE) in acidic media.

| Catalyst/Support | *E*_1/2_  (V) | ECSA (m^2^ g_Pt_^−1^) | MA  (A mg_Pt_^−1^) | SA  (mA cm_Pt_^−2^) | Load Cycle Durability | | | Ref. |
| --- | --- | --- | --- | --- | --- | --- | --- | --- |
|  |  |  |  |  | Conditions | MA retention | SA retention |  |
| Connected Pd@Pt_0.8_/ support-free | 0.92 | 48 ± 3 | 0.59 ± 0.03 | 1.2 ± 0.2 | 0.6–1 V, 60 °C  10k cycles | 70% | 108% | This  work |
| Connected Pt–Fe/ support-free | - | 14 ± 4 | 0.37 ± 0.09 | 2.6 ± 0.3 | 0.6–1 V, 60 °C  10k cycles | 60% | 65% | ^[12]^ |
| Pt-CoO network/ support-free | - | 155.65  ± 4 | 8.37 ± 0.55 | 5.38 ± 0.21 | 0.6–1 V, 80 °C  3.6k cycles | - | 53% | ^[13]^ |
| Pt_3_Cu_97_ network/ support-free | - | 32 | 1.9 | 5.8 | 0.6–1 V, RT  10k cycles | 27% | 31% | ^[14]^ |
| PtCu/Pr_0.15_Ce_0.85_O_2_ | 0.79 | 28.70 | 0.12 | 0.62 | 0.6–1 V, RT  30k cycles | - | 40% | ^[15]^ |
| Pt/W-doped TiO_2_ | 0.786 | 44.1 | 0.038 | - | 0.6–1 V, RT  10k cycles | 97% | - | ^[16]^ |
| Pt/N,C-codoped TiO_2_ | 0.887 | 41.7 | 0.114 | - | 0.6–0.9 V, 25 °C  10k cycles | 83% | - | ^[17]^ |
| Pt/Cr-doped TiO_2_ nanotube | - | 62.7 | 0.221 | - | 0.6–1 V, RT  4k cycles | 61% | - | ^[18]^ |
| Pt/Nb-SnO_2_ | - | 68* | 0.55 | 0.93 | 0.6–1 V, 80 °C  3k cycles | 87% | - | ^[19]^ |
| Pt/Ta-doped SnO_2_ | - | 48 ± 1 | 0.307 | 0.640 | 0.6–1 V, 20 °C  10k cycles | 76% | 90% | ^[20]^ |
| PtCo/Ta–SnO_2_ | - | 54 | 1.4 @ 0.85 V | - | 0.6–1 V, RT  10k cycles | 35% | - | ^[21]^ |
| Pt-SnS_2_/SnO_2_ | 0.895 | 49 | 0.4 | 0.82 | 1.0–1.6 V, RT , 50k cycles | 76% | 70% | ^[22]^ |
| Pt/TiWC (2ML) | - | 60 | 0.420* | 0.690 | 0.4–1 V, RT  10.8k cycles | 78% | 86% | ^[23]^ |
| Pt NRs/GDC | - | - | 0.27 | 0.58* | 0.6–1 V, 80 °C  5k cycles | 87% | - | ^[24]^ |
| Pt/p-BN | 0.902 | 85.21 | 1.06 | 1.24 | 0.6–1.1 V, RT  10k cycles | 93% | - | ^[25]^ |

* Data are from the estimation of the data graph in the literature; RT: Room temperature,

NR: Nanorod, GDC: Gd-doped ceria, BN: Boron nitride.

**References**

[1] T. Tamaki, H. Kuroki, S. Ogura, T. Fuchigami, Y. Kitamoto, T. Yamaguchi, *Energy Environ. Sci.* **2015**, *8*, 3545.

[2] H. Kuroki, T. Tamaki, T. Yamaguchi, *J. Electrochem. Soc.* **2016**, *163*, F927.

[3] B. Akbari, M. Pirhadi Tavandashti, M. Zandrahimi, *Iran. J. Mater. Sci. Eng.* **2011**, *8*, 48.

[4] X. Wang, M. Vara, M. Luo, H. Huang, A. Ruditskiy, J. Park, S. Bao, J. Liu, J. Howe, M. Chi, Z. Xie, Y. Xia, *J. Am. Chem. Soc.* **2015**, *137*, 15036.

[5] A. Ohma, K. Shinohara, A. Iiyama, T. Yoshida, A. Daimaru, *ECS Trans.* **2011**, *41*, 775.

[6] Y. Hashimasa, T. Shimizu, Y. Matsuda, D. Imamura, M. Akai, *ECS Trans.* **2013**, *50*, 723.

[7] S. Li, L. Shi, Y. Guo, J. Wang, D. Liu, S. Zhao, *Chem. Sci.* **2024**, *15*, 11188.

[8] H. Kuroki, T. Tamaki, M. Matsumoto, M. Arao, K. Kubobuchi, H. Imai, T. Yamaguchi, *Ind. Eng. Chem. Res.* **2016**, *55*, 11458.

[9] W. He, J. Liu, Y. Qiao, Z. Zou, X. Zhang, D. L. Akins, H. Yang, *J. Power Sources* **2010**, *195*, 1046.

[10] J. Wang, B. Zhang, W. Guo, L. Wang, J. Chen, H. Pan, W. Sun, *Adv. Mater.* **2023**, *35*, 2211099.

[11] Z. Liu, X. Y. Ling, X. Su, J. Y. Lee, *J. Phys. Chem. B* **2004**, *108*, 8234.

[12] H. Kuroki, Y. Imura, R. Fujita, T. Tamaki, *ACS Appl. Nano Mater.* **2020**, *3*, 9912.

[13] G. W. Sievers, A. W. Jensen, J. Quinson, A. Zana, F. Bizzotto, M. Oezaslan, A. Dworzak, J. J. K. Kirkensgaard, T. E. L. Smitshuysen, S. Kadkhodazadeh, M. Juelsholt, K. M. Ø. Jensen, K. Anklam, H. Wan, J. Schäfer, K. Čépe, M. Escudero-Escribano, J. Rossmeisl, A. Quade, V. Brüser, M. Arenz, *Nat. Mater.* **2021**, *20*, 208.

[14] G. W. Sievers, J. R. Bowen, V. Brüser, M. Arenz, *J. Power Sources* **2019**, *413*, 432.

[15] T. Zou, Y. Wang, F. Xu, *ACS Appl. Mater. Interfaces* **2023**, *15*, 58296.

[16] T. M. Pham, K. Im, J. Kim, *Appl. Surf. Sci.* **2023**, *611*, 155740.

[17] E. Lee, C. Park, D. W. Lee, G. Lee, H.-Y. Park, J. H. Jang, H.-J. Kim, Y.-E. Sung, Y. Tak, S. J. Yoo, *ACS Catal.* **2020**, *10*, 12080.

[18] K.-J. Noh, H. Im, C. Lim, M. G. Jang, I. Nam, J. W. Han, *Chem. Eng. J.* **2022**, *427*, 131568.

[19] G. Shi, T. Tano, D. A. Tryk, A. Iiyama, M. Uchida, K. Kakinuma, *ACS Catal.* **2021**, *11*, 5222.

[20] I. Jiménez-Morales, F. Haidar, S. Cavaliere, D. Jones, J. Rozière, *ACS Catal.* **2020**, *10*, 10399.

[21] K. Kakinuma, M. Hayashi, T. Hashimoto, A. Iiyama, M. Uchida, *ACS Appl. Energy Mater.* **2020**, *3*, 6922.

[22] Z. Lin, J. Liu, S. Li, J. Liang, X. Liu, L. Xie, G. Lu, J. Han, Y. Huang, Q. Li, *Adv. Funct. Mater.* **2023**, *33*, 2211638.

[23] D. Göhl, A. Garg, P. Paciok, K. J. J. Mayrhofer, M. Heggen, Y. Shao-Horn, R. E. Dunin-Borkowski, Y. Román-Leshkov, M. Ledendecker, *Nat. Mater.* **2020**, *19*, 287.

[24] G. Shi, T. Tano, D. A. Tryk, A. Iiyama, M. Uchida, Y. Kuwauchi, A. Masuda, K. Kakinuma, *J. Catal.* **2022**, *407*, 300.

[25] Q. Li, L. Li, X. Yu, X. Wu, Z. Xie, X. Wang, Z. Lu, X. Zhang, Y. Huang, X. Yang, *Chem. Eng. J.* **2020**, *399*, 125827.
